# Cardiovascular risk of dietary trimethylamine oxide precursors and the therapeutic potential of resveratrol and its derivatives

**DOI:** 10.1002/2211-5463.13762

**Published:** 2024-01-21

**Authors:** Chih‐Yao Hou, Yu‐Wei Chen, Sulfath Hakkim Hazeena, You‐Lin Tain, Chang‐Wei Hsieh, De‐Quan Chen, Rou‐Yun Liu, Ming‐Kuei Shih

**Affiliations:** ^1^ Department of Seafood Science, College of Hydrosphere National Kaohsiung University of Science and Technology Taiwan; ^2^ Department of Food Science and Biotechnology National Chung Hsing University Taichung Taiwan; ^3^ Department of Pediatrics Kaohsiung Chang Gung Memorial Hospital Taiwan; ^4^ Institute for Translational Research in Biomedicine Kaohsiung Chang Gung Memorial Hospital Taiwan; ^5^ College of Medicine Chang Gung University Taoyuan Taiwan; ^6^ Department of Medical Research China Medical University Hospital Taichung Taiwan; ^7^ Graduate Institute of Food Culture and Innovation National Kaohsiung University of Hospitality and Tourism Taiwan

**Keywords:** biomarker, cardiovascular diseases, derivatives, resveratrol, TMAO

## Abstract

Overall diet, lifestyle choices, genetic predisposition, and other underlying health conditions may contribute to higher trimethylamine *N*‐oxide (TMAO) levels and increased cardiovascular risk. This review explores the potential therapeutic ability of RSV to protect against cardiovascular diseases (CVD) and affect TMAO levels. This review considers recent studies on the association of TMAO with CVD. It also examines the sources, mechanisms, and metabolism of TMAO along with TMAO‐induced cardiovascular events. Plant polyphenolic compounds, including resveratrol (RSV), and their cardioprotective mechanism of regulating TMAO levels and modifying gut microbiota are also discussed here. RSV's salient features and bioactive properties in reducing CVD have been evaluated. The close relationship between TMAO and CVD is clearly understood from currently available data, making it a potent biomarker for CVD. Precise investigation, including clinical trials, must be performed to understand RSV's mechanism, dose, effects, and derivatives as a cardioprotectant agent.

## Graphical abstract

Genetics and food raise TMAO, which raises cardiovascular risk. This review addresses how resveratrol (RSV) reduces the risk of cardiovascular disease (CVD) via TMAO, discussing sources of TMAO, mechanisms, and metabolism, and highlighting RSV's cardioprotective qualities. TMAO is recognized as a CVD biomarker due to its complicated association with CVD. Further clinical trials are needed to assess RSV's cardioprotective benefits.

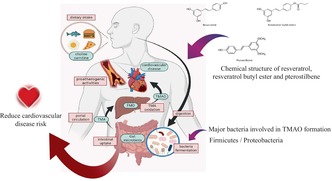

**Abbreviations**
ACC: acetyl‐CoA carboxylase
AMP: adenosine monophosphate
AMPK: AMP‐activated protein kinase
AS: atherosclerosis
BA: bile acid
BPA: bisphenol A
BRCA1: breast cancer type 1
C/EBP: CCAAT/enhancer‐binding protein
CH: cardiac hypertrophy
CLA: conjugated linoleic acid
CVD: cardiovascular diseases
DMA: dimethylamine
DMAP: 4‐dimethyl aminopyridine
EDC: *N*‐ethyl‐*N*′‐(3 dimethylaminopropyl) carbodiimide
FMO: flavin‐containing monooxygenases
FXR: farnesoid X receptor
HDL: high‐density lipoprotein
LDL: low‐density lipoprotein
mPTP: membrane permeability transition pore
NO: nitric oxide
OA: oleic acid
PA: palmitic acid
PPAR: peroxisome proliferator‐activated receptor
RBEs: resveratrol butyrate esters
ROS: reactive oxygen species
RSV: resveratrol
SCFAs: short‐chain fatty acids
SD: Sprague–Dawley
SHRs: spontaneously hypertensive rats
SREBP‐1: sterol regulatory element‐binding protein‐1
TC: total cholesterol
TG: triglyceride
TMA: trimethylamine
TMAO: trimethylamine *N*‐oxide

According to the World Health Organization (WHO), non‐communicable diseases (NCDs) account for 41 million deaths annually, corresponding to two‐thirds of all deaths globally. It is essential to consider that most premature NCD deaths, including cardiovascular diseases (CVD), are reported in economically developing countries [1]. By the next decade, the UN Sustainable Development Goals aim to reduce premature mortality caused by NCDs by a significant level [2]. Early‐stage detection, awareness, and affordable treatment can reduce the risk of avoidable diseases. Biomarkers like trimethylamine *N*‐oxide (TMAO) can be used effectively for early‐stage detection of CVD [3]. Recent studies have established the close relationship between TMAO and prevailing CVD [4]. TMAO is a diet metabolite derived from phosphatidylcholine and l‐carnitine by the action of human gut microbiota. Dietary choline or l‐carnitine in food was metabolized to TMA by gut microbiota, which is converted to TMAO by FMO3 in the liver [5]. TMA is amenable to microbial enzyme trimethylamine dehydrogenase (TMADH)‐mediated degradation into dimethylamine (DMA) and formaldehyde [6]. The kidneys remove most TMAO, and TMAO reductase converts the remainder to TMA [7]. Alteration in the gut microbiota can cause an increase in TMAO levels, thus accelerating the risk of CVD [8].

Diet, as the source of various micronutrients and bioactive compounds, can regulate proatherogenic activities in the human body. Diet modification is a sound strategy for CVD prevention with minimal side effects [9]. Including polyphenolic compounds in the diet can reduce the risk of many chronic diseases, including cancers and diabetes. Polyphenols are interesting bioactive phytochemicals that are of scientific interest, especially with regard to their cardioprotective effects [10]. Resveratrol (RSV) is a crucial polyphenolic compound found in grapes and has been identified to have significant bioactive properties [11]. Several studies have revealed the ability of RSV and its derivatives to reduce circulating TMAO levels and modify the gut microbiota, thereby regulating the risk of CVD [12].

This review aims to present a brief overview of TMAO metabolism in humans and its significance in CVD. It also considers various polyphenol compounds, including RSV and its derivatives in functional foods, as well as their bioactive effects.

## TMAO properties and sources

TMAO (CH_3_)_3_NO is a small amine oxide commonly seen in the tissues of marine organisms. It appears as a colorless water‐soluble solid with a molecular mass of 75.1 Daltons and is usually present in a dihydrate form. The known actions of TMAO in marine organisms are protection against severe marine conditions like high hydrostatic pressure and salinity [13]. Numerous marine species, such as marine elasmobranchs, have tissues that commonly contain TMAO [14]. TMAO is known to provide protection against the negative effects of temperature, salinity, high urea, and hydrostatic pressure in these animals. Small methylated amines including tri‐, di‐, and monomethylamine, which are precursors of marine aerosols and the greenhouse gas nitrous oxide in the marine atmosphere, can be produced by the metabolism of TMAO [15]. Besides these known roles, the significance of TMAO has been elucidated recently, especially as an indicator of CVD in humans.

TMAO is generated within organisms through the metabolic pathway of common food constituents, including carnitine, choline, and betaine, by gut microbiota. This pathway initially results in the formation of trimethylamine (TMA), which is then oxidized by flavin‐containing monooxygenases, an enzyme group located in the host liver [16]. FMO3 and FMO1 are the hepatic enzymes responsible for converting TMA to TMAO. FMO3 is the primary enzyme that catalyzes the conversion because FMO1 has a very low specific activity. Abnormalities in FMO3 function can result in the impaired conversion of TMA to TMAO. Elevated systemic TMA is excreted through urine, sweat, and breath leading to a rare autosomal recessive disorder called trimethylaminuria, marked with a strong fish‐like odor [17].

**Fig. 1:**
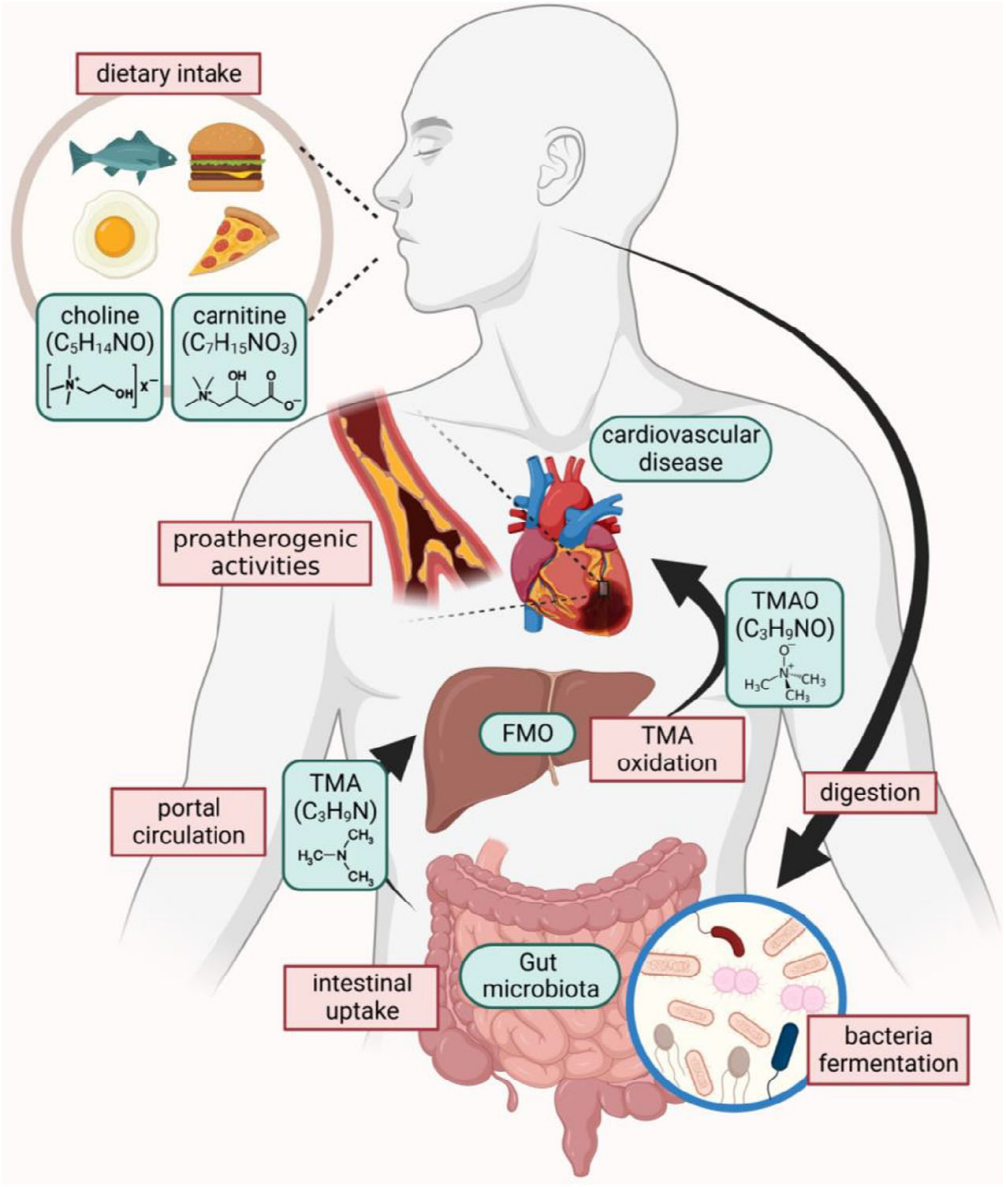
Overall pathway of TMAO‐induced CVD and the chemical formula of choline, carnitine, and TMA, TMAO.

Average human blood plasma TMAO titers in healthy individuals are 3 μmol·L^−1^, but are nearly 40 μmol·L^−1^ in patients with renal failure [18]. Preclinical experiments revealed that TMAO could directly affect heart health by inducing myocardial hypertrophy and fibrosis, endothelial cell and vascular inflammation, and cardiac mitochondrial dysfunction, exasperating the advancement of CVD [7]. Platelet activation and thrombus formation are also important causes of CVD induced by TMAO. The gut microbe‐dependent metabolite TMAO directly increases the likelihood of platelet hyperreactivity and thrombosis *in vivo*, which are important risk factors for developing cardiometabolic disease complications such as stroke and heart attack [19]. The pro‐atherogenic activity of TMAO can be triggered by inducing inflammation of the vascular wall, inducing reactive oxygen species (ROS) production, and impairing cholesterol reverse transport [20]. The properties and sources of TMAO are shown in Fig. 1.

### TMAO biosynthesis

TMAO biosynthesis has been influenced by three major factors: dietary precursors, gut microbiota, and TMA oxidation [21].

#### Diet

Diet is a primary component in generating TMAO in the human body. Red meat, eggs, dairy products, and salt‐water fish are rich sources of choline and carnitine, while betaine is generally of plant origin [20]. Besides the dietary precursors, TMAO is naturally present in fish and can be absorbed during digestion and excreted through urine [22]. Since its discovery in marine fish and crustaceans, TMAO has been linked to decreased osmoregulatory expenses, increased buoyancy, and the prevention of pressure‐induced protein instability [23]. Gawrys‐Kopczynska *et al*. [22] reported that deep‐sea organisms build up TMAO under physiological settings to shield enzyme activity, such as lactate dehydrogenase (LDH), from hydrostatic and/or osmotic pressure stress. Consumption of higher amounts of red meat in the diet enhances the risk of CVD by increasing the production of TMAO [24]. A recent study showed that long‐term consumption of red meat elevated TMAO levels [25]. Another study of 84 136 women showed that higher consumption of red meat remarkably increased the risk of CVD [26].

Plasma TMAO levels depend on many factors, including direct intake of TMAO from the diet (fish, seafood), intake of substrates (choline, carnitine) available to TMA‐producing bacteria, the activity of intestinal microbiota, TMAO intestinal absorption, the permeability of the intestinal‐blood barrier to TMA, hepatic metabolism of TMA (oxidation of TMA to TMAO), and finally absorption by extrahepatic tissues or excretion through the kidneys [27]. Significantly more free TMAO was present in seafood than was produced by gastrointestinal microorganisms from choline and carnitine in red meat and eggs. Fish make 46–62 times more TMAO than eggs or beef [28], but fish consumption reduces the risk of cardiovascular disease. Even low‐fat fish (cod) consumption can reduce cardiovascular disease risk factors [29], which seems to be a paradox. Kruger and coworkers noticed that fish consumption was correlated with increased plasma and urine TMAO concentrations, while meat consumption was only linked with plasma TMAO concentrations. Cho *et al*. [28] reported that in a feeding experiment with healthy young males, a substantial amount of TMAO was detected in plasma 15 min after fish ingestion, indicating that dietary TMAO can be assimilated without being processed by gastrointestinal microorganisms. The studies showed a strong association between the nature of diet and TMAO levels in the human body.

#### Gut microbiota

Gut microbiota are the next important element in the synthesis of TMAO, and alterations in the gut microbiota have remarkable effects on TMAO levels. Intestinal bacteria typically have salient functions such as nutrient degradation, absorption, and immune system stimulation [30]. In addition to metabolic and immune functions, gut microbiota also indicates various health conditions. Alteration in gut microbiota has been known to be connected with various diseases, such as obesity, diabetes mellitus, digestive system diseases, oxidative stress, and cancers [31].

The key source of TMAO in the blood is gut bacteria activity. Reduced concentration of TMAO in antibiotic‐treated human and laboratory animals confirmed that gut microbiota is the primary source of TMAO in blood. A near non‐detectable amount of TMAO was observed in body fluids after a week of treatment with antibiotics, pointing out that the gut microbiome disruption affects TMAO synthesis [32]. It was found that antibiotics could suppress TMAO synthesis by inhibiting the gut microbiota's conversion of l‐carnitine to TMA.

Firmicutes and Proteobacteria are the primary bacteria responsible for producing TMA in the gut from choline and carnitine‐containing food sources. Studies showed that the bacterial families associated with TMA/TMAO production are Deferribacteraceae, Anaeroplasmataceae, Prevotellaceae, and Enterobacteriaceae. Recently eight bacterial strains involved in TMA formation, including two different phyla (Firmicutes and Proteobacteria), have been identified (Table 1) [33].

**Table 1:** Eight species spanning two phyla (Firmicutes and Proteobacteria), and six genera demonstrated considerable choline consumption and TMA accumulation.

Class	Bacterial isolates^a^
Firmicutes	*Anaerococcus hydrogenalis*
Firmicutes	*Clostridium asparagiforme*
Firmicutes	*Clostridium hathewayi*
Firmicutes	*Clostridium sporogenes*
Proteobacteria	*Escherichia fergusonii*
Proteobacteria	*Proteus penneri*
Proteobacteria	*Providencia rettgeri*
Proteobacteria	*Edwardsiella tarda*

^a^ Only choline‐supplemented media produced TMA from these strains.

CutC and CntA are the key genes responsible for carnitine metabolism, coding for Choline‐TMA lyase and carnitine oxygenase enzymes, respectively [34]. Betaine is metabolized by the enzyme betaine reductase, which is coded by the GrdH gene. In mammals, CutC is the predominant gene in omnivores and carnivores, but the CntA gene is absent from herbivores [30]. The choline‐degrading sulfate‐reducing bacterium *Desulfovibrio desulfuricans* harbors a gene cluster (including cutC and cutD) responsible for the breakdown of choline, which will result in TMA formation [35]. Acinetobacter and Serratia, recognized as possessing CntA and CntB genes, encode the two subunits of the oxidoreductase enzyme accountable for TMA synthesis from l‐carnitine. Moreover, YeaW/YeaX are another gene pair that encode some oxygenase and oxidoreductase with substrate specificity for choline, betaine, and carnitine and carry out the conversion of respective substrates to TMA. [16]. These genes, along with the orthologs and homologs, can be seen in broad genera of gut bacteria, including Gammaproteobacteria (*E. coli*, Citrobacter, *Klebsiella pneumoniae*, Providencia, and Shigella), Betaproteobacteria (Achromobacter), Firmicutes (Sporosarcina), and Actinobacteria [36]. The Firmicutes/Bacteroidetes ratio has been used to study TMAO levels [24]. Metagenomic analysis of intestinal microbiota revealed that the ratio of Firmicutes to Bacteroidetes in subjects with atherosclerosis (AS) is higher than compared to the control [37].

Besides functional genes, the intestinal microenvironment also affects TMAO production. Yoo *et al*. [38] found that prolonged high‐fat diets affect intestinal epithelial function and enhance *E. coli* choline catabolism. High‐fat diets disrupt mitochondrial bioenergetics in colonic epithelial cells, increasing luminal oxygen and nitrate bioavailability and *Escherichia coli*'s respiratory‐dependent choline catabolism. *E. coli* choline catabolism increased TMAO, a potentially harmful gut microbiota metabolite [38]. Thøgersen and coworkers carried out a study on the effect of red and white meat consumption and TMAO concentration and its relationship with the gut microbiome. The expression of genes linked to TMAO production was not affected by diet alone. The results indicated that diet‐induced TMAO formation is not due to changes in gene expression, but primarily caused by modification of the gut microbiota [39].

Nutritional, metabolic, or genetic factors cause these disparities between men and women. The degree of obesity may influence changes in the gut microbiota between men and women, as suggested by Haro *et al*. [40]. The identification of gender variations in the incidence of metabolic and intestinal inflammatory illnesses may be influenced mainly by the documented divergence in gut microbiota between men and women [40]. According to Dehghan *et al*. [41], a meta‐analysis showed a favorable correlation between circulating TMAO and obesity as indicated by an elevated BMI. Additionally, it was found that in those who appeared to be in good health, there was a dose‐dependent correlation between circulating TMAO and obesity [41].

#### TMA oxidation

Research has demonstrated that human exposure to TMA can result in eye [42] and skin [43] discomfort. Furthermore, there have been reports of reproductive and developmental harm [44]. TMA, but not TMAO, was discovered to be toxic to vascular smooth muscle cells (VSMCs) [45], and a conference article stated that TMA, but not TMAO, was vasoconstrictive [46]. Jaworska *et al*. [47] reported that TMA exerts toxic effects on the cardiovascular system. After absorption, most TMA (nearly 95%) is oxidized to TMAO, which is transported to the tissues for accumulation as an osmolyte or, more frequently, cleared by the kidneys [15], which is then excreted, mainly with urine in a 3 : 95 TMA : TMAO ratio within 24 h. Consequently, because of the difference in concentration, the physiological effects of TMAO are more widely appreciated. Specifically, intravenous administration of TMA to anesthetized rodents increased blood pressure, and *in vitro* studies revealed deleterious effects [45]. Another critical parameter that affects TMAO formation is the oxidation of TMA. TMA is a precursor of TMAO, which is transported to the liver and oxidized by flavin‐containing monooxygenases (FMO). Among the FMO oxidizing enzymes, Vitamin B2‐dependent FMO3 exhibits the highest specific activity. FMO3 is the critical player in TMAO formation, as FMO3 hepatic knockdown mice showed TMAO levels lower than normal mice. Along with this, gender plays a vital role in the expression of FMO3. In mice, FMO3 was lowered because of testosterone [48]. Another study also concluded that female mice showed increased plasma TMAO levels and hepatic FMO3 activity as compared to male mice [33]. However, the differences in FMO3 expression are moderate, primarily because of diet variation in humans.

### TMAO and cardiovascular disease

Higher TMAO levels raise the risk of adverse cardiovascular events. Overall dietary habits, lifestyle decisions, genetic predisposition, and other underlying medical disorders may elevate TMAO levels and increase risk of cardiovascular disease. In the last decade, elevated blood TMAO levels have been identified as a biomarker of elevated risk of CVD. Various researchers have observed and reported a positive relationship between the circulating TMAO level and the risk of CVD [3, 49].

Wilson *et al*. [50] believed elevated TMAO levels increase the risk of CVD mortality by 2.5‐fold. Among CVD, AS is considered to be the most lethal. Generally, high serum cholesterol and blood pressure, family history, obesity, smoking, and a high‐fat diet are the significant risk factors for atherogenesis. The relationship between elevated TMAO levels and AS was identified, and it was hypothesized that TMAO can generate inflammatory reactions in the vascular wall, induce reactive oxygen species (ROS), impair cholesterol reverse transport, and may induce atherogenesis. Koeth *et al*. [32] also reported that TMAO moderated cholesterol metabolism to accelerate the progression of AS. Jang and Lee [51] reported that intestinal microbiota generates TMAO in high‐calorie, high‐sugar diet rats, decreasing glucose tolerance, suppressing hepatic insulin signaling, and increasing adipose tissue inflammation.

In the wake of the above findings, there has been substantial research looking to connect TMAO with CVD. The association between elevated TMAO concentrations and a 62% increased risk of all‐cause mortality was determined by a recent systematic review and meta‐analysis of 19 prospective studies (*n* = 19 256, including 3315 incident CVD cases). On the contrary, those who had significantly higher levels of TMAO precursors (l‐carnitine, choline, or betaine) were only 1.3 to 1.4 times more likely to experience severe adverse cardiovascular disease events than those who had low levels [52]. An additional meta‐analysis, comprising a total of 10 245 patients and 11 prospective studies, discovered that increased circulating TMAO was linked to a 55% increased risk of all‐cause mortality and a 23% increased risk of cardiovascular events [53]. Nevertheless, the relationship between TMAO and CVD remained tenuous, and not all investigations discovered a substantial correlation [53]. The methods through which TMAO might enhance the risk of CVD are, in fact, unclear and include a wide variety of clinical diseases. Not all studies have shown a link between TMAO and CVD (Table 2). In a study of patients undergoing coronary angiography, Mueller *et al*. [54] discovered that patients with diabetes had greater plasma concentrations of TMAO than controls with euglycemia. Although high levels of TMAO in the blood have been linked to CVD, eating seafood, which is naturally high in TMAO, is usually seen to be beneficial to one's health.

**Table 2:** Effects of TMAO on cardiovascular‐related diseases under different health conditions.

Health condition	Duration of study	Impression	References
Overweight/obese women pre‐screened for insulin resistance and/or dyslipidemia^a^	Eight‐week controlled feeding trial	Did not improve endothelial function or reduce plasma TMAO	[159]
Seventy‐eight patients that participated in the atrial fibrillation (AF) risk study^b^	Two‐week event recorder at baseline and 1‐year follow‐up	Higher levels of TMAO are associated with more progressed forms of AF	[160]
Two hundred and sixty‐two symptomatic peripheral artery disease (PAD) patients^c^	PAD patients were followed up for a mean period of 4 years (min 1 max 102 months) every 3, 6 or 12 months	Patients with TMAO >2.26 μmol·L^−1^ exhibited higher risk of cardiovascular death	[4]
Three hundred and eleven individuals with T2D and albuminuria^d^	The trial ended the intervention after an average of 7.8 years. Thereafter, observational studies continued	Higher choline, carnitine and the weighted sum score of the four metabolites were associated with higher risk of decline in eGFR	[161]
Major adverse cardiovascular events (MACE) in coronary heart disease (CHD) patients^e^	Follow‐up ≥4 years	TMAO concentrations increased; 58% higher risk of MACE in patients with CHD	[162]

^a^ Endothelial function, plasma TMAO concentration, and CVD risk.

^b^ Gut microbiome, plasma TMAO concentrations, and AF risk.

^c^ Gut bacterial function, plasma TMAO concentration, renal function, and PAD risk.

^d^ T2D and albuminuria, plasma TMAO concentration, worsening renal function and CVD.

^e^ Meta‐analysis, plasma TMAO concentration and CHD risk.

Some theories suggest that eating high‐TMAO fish affects CVD. Fish is high in omega‐3 fatty acids, which are good for the heart. Omega‐3 fatty acids decrease triglycerides, improve cardiac function, lower blood pressure, and fight vascular inflammation. These benefits may offset TMAO's cardiovascular risks [55]. Other chemicals in fish may reduce TMAO's negative effects. Some fish may include antioxidants, anti‐inflammatories, or other bioactive compounds [56] that may reduce TMAO damage. The manner in which fish is cooked may potentially affect cardiovascular health [57]. Studies have demonstrated that cooking methods may affect food TMAO concentration and CVD risk [58]. Overall, eating fish with high TMAO levels has relatively few adverse effects on CVD, even though there is a relationship between TMAO and CVD. Other beneficial components in fish and cooking techniques may explain this. To better grasp this problem, further research is necessary, as this is still an active field of study.

#### Relationship between TMAO and atherogenesis

The precise mechanism through which TMAO stimulates atherogenesis is unknown. Alterations in cholesterol synthesis, foam cell formation stimulation, and bile salt metabolism changes have been hypothesized to be the underlying processes. The atherogenic capability of TMAO is induced by blocking hepatic bile acid (BA) production [15]. Koeth *et al*. [32] discovered that TMAO is linked to changes in cholesterol metabolism, which helps advance CVD.

Wang *et al*. [59] reported that a diet with TMAO precursors could increase macrophage scavenger receptors associated with AS. Furthermore, the administration of dietary TMAO to mice resulted in a substantial decrease in the absorption of cholesterol and the expression of the bile acid synthetic enzymes cytochrome P450, family 27, subfamily a, polypeptide 1 (Cyp7a1) and cytochrome P450, family 27, subfamily a, polypeptide 1 (Cyp27a1) in the liver [32]. Cholesterol elimination is heavily reliant on the bile acid pathway, and thus inhibiting this pathway may promote atherogenesis. TMAO‐treated mice exhibited decreased levels of bile acid transporters (Oatp1, Oatp4, Mrp2, and Ntcp) and bile acid synthetic enzymes (Cyp7a1 and Cyp27a1) in the liver [32]. TMAO induces the expression of inflammatory cytokines and adhesion molecules in human and mouse studies. According to Zhu *et al*. [60], TMAO produced by gut microbes can enhance platelet reactivity and thrombosis. Clinical investigations have also found a relationship between plasma TMAO levels and thrombosis episodes. By regulating intracellular Ca^2+^ release, the direct reaction of platelets to TMAO increases platelet aggregation [19].

Likewise, a clear link exists between oxidative stress with CVD. Inflammation and the buildup of reactive oxygen species (ROS) begin the development of atherosclerotic plaques and culminate in the creation of oxidized LDL. Finally, oxidized LDL accumulates in endothelial walls, leading to the development of atherosclerotic plaques [10]. Collectively, these findings provide a possible link between gut bacteria, platelet activation, and the risk of thrombosis. More research is required to show the function of TMAO in the formation of CVD [15].

#### TMAO and bile acid metabolism

Bile acid production is the principal method for removing excess cholesterol in the body. Bile acid changes have been linked to inflammation and the development of AS. Dietary choline has been shown to lower the amount of bile acid in mice [20]. In addition, mice with increased TMAO levels exhibited a substantial decrease in cholesterol absorption and liver expression of the bile acid‐synthesizing enzymes cytochrome P450, family 7, subfamily a, and polypeptide 1 and 21 (Cyp7a1) (Cyp27a1), as well as bile acid transporters [32].

By suppressing the activation of the farnesoid X receptor (FXR), TMAO suppressed hepatic bile acid production and altered the bile acid synthesis pathway [20]. When bile acids activate FXR, it causes dramatic changes in bile acid balance and the transcription of genes involved in bile acid production. The binding of FXR to bile acid determines the degree of bile acid‐induced FXR activation. FXR binding causes bile acid export mechanisms in the small intestine and liver to be upregulated while bile acid transport systems are downregulated [50].

Recent research on the inhibitory action of naturally occurring chemicals in food against microbial choline TMA‐lyase may be helpful in the medical field [61]. Flavonoid‐rich plant extracts can decrease AS progression, potentially by lowering serum TMAO levels and boosting BA elimination [62]. Although RSV has several biological activities, including protecting against CVD [63] and regulating gut bacteria [64], direct studies on the effect of RSV on TMAO are scarce [12, 61, 65, 66]. Since the production of TMAO is related to the type of food intake [25, 28], the growth and decline of intestinal bacteria [16] and the proatherogenic activity of TMAO may be caused by inducing inflammation of the vascular wall, inducing reactive oxygen species (ROS) production, and impairing cholesterol reverse transport [20]. Therefore, judging from the above‐mentioned TMAO pathogenic pathways, RSV does have the potential to affect the TMAO content in organisms. For example, RSV may decrease the possibility of many diseases through gut microbiota‐modifying therapy [67, 68, 69]. Therefore, eating more polyphenol‐rich foods is a beneficial strategy to prevent TMAO‐induced CVD [70]. For example, several studies have revealed the ability of RSV and its derivatives to reduce circulating TMAO levels and alter gut microbiota, ultimately modulating CVD risk [1, 2, 3]. As such, more studies into the impact of RSV and its modified derivatives on lowering the severity of atherogenic episodes is required.

## Application potential of phytochemicals such as polyphenols, RSV and its modified derivatives in regulating CVD

TMAO is a unique and independent risk factor for developing AS, partly through suppression of hepatic bile acid production. The role of gut microbiota is important in the pathogenesis of TMAO‐induced AS. RSV was shown to attenuate TMAO‐induced AS as a natural polyphenol with prebiotic advantages. A growing body of research supports the idea that phenolic phytochemicals with low bioavailability may exert their primary biological effects by altering the gut microbiota. A recent study showed that RSV decreased TMAO levels, thereby reducing TMAO‐induced AS and promoting gut microbiota remodeling through hepatic BA neosynthesis [12]. Although Annunziata *et al*. [65] speculated that polyphenols could donate electrons to TMAO, and TMAO acts as an electrophile, leading to the reduction of TMAO and the production of TMA, such a hypothesis has not been proven *in vivo* or confirmed experimentally.

When studying TMAO levels and their relationship to cardiovascular disease, it is important to consider the different dietary factors that may influence them. In this context, RSV and other compounds found in the plant have been the subject of research to assess whether they affect TMAO levels or benefit cardiovascular health. In addition, many other plant compounds can affect TMAO levels or cardiovascular health, and some examples include anthocyanins in berries, catechins in green tea, and anthocyanins in blueberries [71]. In conclusion, eating plants in general affects TMAO levels. RSV is just one of several plant compounds studied to assess its potential impact on TMAO levels and cardiovascular health. However, although RSV is not the only plant compound that can affect TMAO levels or cardiovascular health, RSV has a wide range of biological functions, including antioxidant activity, CVD prevention, and control of gut bacteria, which contribute to its association with the proatherogenic activity of TMA and TMAO produced by gut bacteria. For example, causing inflammation in the arterial wall leads to the formation of ROS and reduces the function of the anti‐cholesterol transport system. In this way, this study investigated the potential therapeutic ability of RSV to prevent CVD and alter TMAO levels. In addition, the impact of RSV and its modified derivatives on intestinal bacteria and the potential application of esterified derivatives are specifically discussed here.

On the other hand, how polyphenols, including RSV and RSV‐related derivatives, can directly or indirectly reduce the content of TMA and TMAO in organisms still occupies a huge research space for future exploration. In other words, investigating the anti‐oxidation, anti‐obesity, regulation of intestinal bacteria and any other biological activity potential of RSV and RSV derivatives similar to polyphenols, especially pterostilbene, is still of considerable interest. Related studies are described below.

### TMAO‐lowering phytochemicals

It is critical to consider several dietary components when analyzing the link between TMAO levels and cardiovascular disease. In this respect, studies have concentrated on determining whether RSV and other substances derived from plants can influence TMAO levels or have cardiovascular advantages. Some phytochemicals, like phenolic molecules, carotenoids, and phytosterols, have a wide range of bioactivities that can help avoid or treat CVD [72]. At least some of the heart health benefits of eating fruits and vegetables have been linked to their phytochemicals [72].

This review pays attention to those compounds that have been shown to reduce TMAO formation, as well as phenols and their derivatives that can potentially prevent TMA formation, as TMAO may be involved in one of the multiple mechanisms by which they prevent the development of atherosclerosis. Using phytochemicals to reduce TMAO formation can be considered a dietary intervention. Since the discovery of TMAO as a pro‐atherogenic metabolite is relatively recent, only relatively small numbers of polyphenol‐rich extracts and single phenolic compounds have been investigated as TMA/TMAO lowering agents [73]. Since polyphenols generally have low bioavailability, these compounds reach high concentrations almost exclusively in the gut. Thus, such properties of polyphenols make them good candidates for inhibiting TMAO formation in terms of modulating gut microbiota and/or inhibiting the function of TMA‐lyases (TMA lyases) [74].

Several plant extracts rich in phenolic compounds have been shown to reduce TMAO. For example, extracts from the fruit of Bokbunja (Rubus coreanus Miquel) demonstrate TMAO‐reducing properties [75]. Administration of anthocyanin‐rich blue honeysuckle berry extract to diet‐induced hypercholesterolemic male Sprague–Dawley (SD) rats improved serum dyslipidemia and decreased serum TMAO levels [76]. Polymethoxyflavones cause a decrease in hepatic FMO3 expression, modulation of the relative abundance of Firmicutes and Bacteroidetes in the gut, and other anti‐atherogenic effects [77]. Bresciani *et al*. [78] assessed the potential of different polyphenol‐rich sources for TMA formation. The results showed that differences in the initial substrate (choline or l‐carnitine) and the microbiota source (vegan or omnivorous) influenced the differential effects of polyphenol sources on TMA formation.

In addition, curcumin has broad cardioprotective functions [79], but its TMAO‐lowering effect is controversial [80]. Shi *et al*. [81] reported that the alkaloids berberine and trigonelline have anti‐atherogenic properties associated with their ability to reduce TMAO formation *in vivo*. Berberine appears to reduce TMAO formation by modulating gut microbiota composition and possibly function, and by reducing host FMO3 gene expression [82]. Anwar *et al*. [83] recently demonstrated the potential of trigonelline to reduce TMAO‐induced atherosclerosis and its relationship to the gut microbiota in female C57BL/6J mice. Glucosinolates in Brussels sprouts may exert anti‐atherogenic functions [84]. Cashman *et al*. [84] demonstrated the potential of glucosinolate‐rich Brussels sprouts to inhibit human FMO3. Butler and Fenwick [85] found that goitrin inhibits the oxidation of TMA to TMAO by FMO3 by competing with the enzyme active center.

Allicin is a volatile thiosulfinate found in garlic, onion, and shallot, with relevant biological effects [86]. Wu *et al*. [87] recently demonstrated that allicin reduced plasma TMAO levels to healthy control concentrations in l‐carnitine‐supplemented C57BL/6J mice. Wu *et al*. [88] reported that lycopene administration normalized serum TMAO in male C57BL/6J mice fed a high‐fat diet. This effect was accompanied by the normalization of hepatic FMO3 activity and modulation of gut microbiota. Phytosterols may help lower TMAO levels in the body. For example, in a study by Ryan *et al*. [89], the administration of phytosterol esters to mice fed a high‐fat, high‐cholesterol diet resulted in decreased levels of TMA in fecal samples from the mice.

Iglesias‐Carres *et al*. [72] speculated that phytochemicals with reduced TMAO bioactivity might act as prebiotic compounds by (a) modulating gut microbiota composition and/or function; (b) directly inhibiting TMA lyase; or (c) inhibiting Hepatic FMO3 expression or activity. Although the TMA and/or TMAO‐lowering properties of phytochemicals are cardioprotective, other associated cardioprotective effects have also been reported for these phytochemicals. Thus, the cardioprotective function of phytochemicals may arise from a combination of different mechanisms, including TMAO‐reducing properties.

### Polyphenols, RSV and RSV and its modified derivatives modify the gut microbiota

Polyphenols are the most prevalent organic compounds derived from plants. More than 8000 polyphenols have been identified. The distribution of polyphenols varies among tissues and subcellular components. Generally, insoluble polyphenols are found in vacuoles [90]. Polyphenols are believed to protect against acute and chronic diseases such as type 2 diabetes, obesity, cancer, and CVD [91]. Studies showed that polyphenols could significantly regulate gut microbiota, thereby modulating their bioactive properties. Mehmood *et al*. [92] investigated the cardioprotective effects of olive polyphenols. Blueberry extract polyphenols can increase the bifidobacteria, while green tea extract can balance *Clostridium difficile*, *Escherichia coli*, and *Salmonella typhimurium* [93]. Prominent evidence suggests that polyphenols may promote the beneficial actions of probiotics [91]. Polyphenols can modify gut microbiota, but their bioavailability varies with chemical structure. Many studies claim such bioactive properties are exerted by altering the gut microbiota [94].

The literature on gut microbiota and obesity reported that RSV could significantly increase the fasting‐induced expression of circulating lipoprotein lipase inhibitor (lipoprotein lipase inhibitor) [95]. Zhao *et al*. [96] confirmed that combining quercetin and RSV can affect the intestinal microbiota and thus produce anti‐obesity effects. Combining quercetin and RSV can significantly increase the production of Bacteroidales_S24‐7_group, Christensenellaceae, Akkermansia, Ruminococcaceae and Ruminococcaceae_UCG‐014, and Ruminococcaceae_UCG−005. Similar studies have also found that RSV can normalize intestinal microbiota and achieve anti‐obesity effects by reducing Lactococcus, Clostridium XI, Oscillibacter, and Hydrogenoanaerobacterium [97]. A large number of studies have confirmed that intestinal microbiota and obesity are closely related to host metabolic abnormalities [98], and the ratio of Firmicutes/Bacteroidetes (F/B ratio) associated with obesity is lower in obese individuals than in non‐obese individuals [99].

Pterostilbene, structurally related to RSV, reduced TMAO plasma levels in female C57BL/6J mice supplemented with carnitine and also had other anti‐atherogenic effects [100]. Chen *et al*. [12] found that RSV reduced choline‐induced aortic plaque in C57BL/6J ApoE^−/−^ mice by lowering TMAO. A potential mechanism of action for RSV to reduce TMAO effects is the modulation of TMA‐producing bacteria. Etxeberria *et al*. [101] found that treatment of Wistar rats fed on a diet high in fat and sugar with RSV altered the microbiota. Supplementing with RSV increased the number of *Lactobacillus*, *Bifidobacterium*, *Prevotella*, *Helicobacter*, *Bacteroides*, and other unclassified bacterial groups [12]. Several bacterial taxa were also linked to changes in plasma TMA levels. Additionally, it is known that circulating TMAO and aortic lesions are reduced in many types of antibiotic‐induced microbiota suppression. [32, 59].

In addition, RSV butyrate esters (RBEs), synthetic RSV derivatives, reduced adiposity in female SD rats, as reported in our previous study [102]. Since RBEs can significantly modify the gastrointestinal microbiota (decrease the Firmicutes/Bacteroidetes (F/B) ratio) in the progeny of female SD rats, we concluded that bisphenol A (BPA) would delay body fat metabolism in female offspring and cause obesity. RBEs normalized lipid metabolism and intestinal microbiota in female progeny.

From the above, it can be seen that a comprehensive understanding of the interaction between RSV and gut microbiota will not only provide insights into the pharmacological effects of RSV, but also may lead to the development of gut microbiota‐modifying therapy [67, 68, 69].

### Cardioprotective effects of RSV

The observation that TMAO is one of the risk factors for cardiovascular disease (CVD) suggests that RSV can mitigate its deleterious effects and prevent the pathogenesis of CVD. In addition, RSV can improve endothelial function, left ventricular diastolic function, and endothelial function, and also lower LDL‐cholesterol level and fibromuscular dysplasia (FMD). In brief, along with modifying the gut microbiota, RSV significantly changes the markers associated with CVD (Table 3). RSV affects blood pressure regulation, vascular activity, ischemia–reperfusion damage, platelet aggregation inhibition, AS prevention, and cardiac hypertrophy (CH). Numerous studies have demonstrated the advantages of RSV, which are covered in the following section.

**Table 3:** Effects of RSV as a cardioprotectant agent.

Conditions of subjects	RSV dosage	Impression after RSV administration	References
Twenty‐four hypertensive patients between 45 and 65 years old with baseline endothelial dysfunction	A single dose of trans‐RSV (300 mg)	An improvement in endothelial function, especially in women and those with higher LDL‐cholesterol	[163]
Forty patients with myocardial infarction. Randomly divided into two groups	A 10 mg RSV capsule or placebo daily for 3 months	Improved left ventricular diastolic function and endothelial function, lowered LDL‐cholesterol level and protection against unfavorable hemorheological changes measured in patients with coronary artery disease (CAD)	[164]
Twenty‐eight obese but otherwise healthy adults (BMI: 33.3 ± 0.6 kg·m^−1^)	A 75 mg capsule of trans‐RSV (Resvida) or placebo daily for 6 weeks	Fibromuscular dysplasia (FMD) improvements significant after RSV administration	[165]

#### Vascular activities and blood pressure regulation

A meta‐analysis by Fogacci *et al*. [103] suggests that RSV can actively reduce increased blood pressure in diabetic patients when provided as daily doses. Another meta‐analysis of 247 subjects produced consistent results, showing that RSV can reduce high blood pressure at higher doses while having no effects in lower doses [104]. RSV has a dose‐dependent influence on blood pressure in animal studies. In combination with hydralazine, RSV at smaller dosages was similarly more efficacious than RSV or hydralazine alone. As a result, RSV can be used as a supplement to treat high blood pressure. In a human investigation with six randomized control trials, RSV intake at a dosage of 150 mg·day^−1^ lowered systolic blood pressure [105].

#### Inhibition of platelet aggregation

Activation of platelet aggregation is the primary event in the development of atherothrombosis. Hence, inhibition of platelet aggregation is essential to prevent thrombotic events. A study showed inhibition of platelet adhesion to collagen during treatment with RSV, thus controlling thrombin‐induced platelet aggregation [106]. Human platelets stored with RSV for 5 days released less thromboxane B2 and prostaglandin E2, which are essential factors in blood clot formation, as compared to control platelets [107]. Marumo *et al*. [108] observed that thapsigargin‐induced Ca^2+^ entry into platelets and subsequent platelet aggregation was significantly inhibited by RSV at 6.25 μm or higher concentrations.

Other mechanisms involved in the inhibition of platelet aggregation include inhibition of the MAPK pathway, activation of the nitric oxide/cGMP pathway, and inhibiting phosphoinositide signaling [109]. The administration of grape juice *ex vivo* reduced platelet aggregation [110]. Even though different mechanisms can explain platelet aggregation inhibition, it is widely accepted that RSV can act as a critical factor in platelet aggregation inhibition and thus prevent the progression of atherogenic plaques.

#### Ischemia–reperfusion injury

*In vivo* and *in vitro* experiments demonstrated that RSV can potentially reduce oxidative stress and Fe^2+^ content. RSV reduces oxidative stress and ferroptosis, which protects against cardiac ischemia–reperfusion damage [63]. RSV protects newborn cardiomyocytes against ischemia–reperfusion damage by lowering intracellular calcium, inhibiting apoptosis, and increasing the activity of reactive oxygen species‐scavenging enzymes such as superoxide dismutase. Modulation of the mitochondrial membrane permeability transition pore (mPTP), activation of adenosine monophosphate (AMP)‐activated protein kinase (AMPK), and stimulation of nitric oxide synthase are all possible pathways for RSV's antioxidant actions. [111]. Yang *et al*. [112] demonstrated that RSV could attenuate myocardial ischemia–reperfusion injury via activation of the VEGF‐B/antioxidant signaling pathway. It is clear from the above data RSV efficiently reduces ischemia–reperfusion injury.

#### Atherosclerosis

AS, accumulation of lipids on the vessel wall, is the pathological foundation for many cases of CVD and the primary reason for significant cardiovascular deaths [113]. The buildup of low‐density lipoprotein (LDL) in the artery wall is the molecular mechanism underpinning AS. LDL oxidation, in addition to deposition, is essential in atherogenesis. Oxidized LDL increases inflammation, which leads to plaque formation on the vessel wall. As a result, LDL oxidation control is a critical anti‐atherosclerotic treatment target. Thrombosis is another cause of acute coronary syndrome; AS is the primary root problem.

RSV treatment ameliorated the thickening of the coronary artery wall and reduced regions of atherosclerotic lesions on the aorta by preventing the accumulation of serum lipids such as TC (total cholesterol) and TG (triglyceride). RSV was discovered to limit platelet activation, vascular smooth muscle cell growth, and lower the production of cell adhesion molecules, monocyte colony‐stimulating factors, matrix metalloproteinases, and growth regulators. It decreased the thresholds of advanced glycation end products and their receptor in vascular tissue, decreased serum total cholesterol and triglycerides (TG), and raised high‐density lipoprotein (HDL) cholesterol. In mouse tests, RSV lowered the amount of AS [114]. RSV reduced lipid buildup, regulated gene expression linked to lipogenesis and lipolysis, and modified vascular function. RSV also protects vascular endothelial cells from oxidized LDL‐induced apoptosis [115]. RSV has shown beneficial effects against CVD such as protection against AS, hypertension, heart failure, diabetes, obesity, and aging. It has been hypothesized that RSV induces vasodilation through nitric oxide (NO) production and reduces platelet aggregation [115]. The cardioprotective effects of RSV are believed to be related to its preconditioning‐like action. During preconditioning, small doses of RSV can exert an adaptive stress response, inducing the expression of cardioprotective genes, such as genes responsible for heat shock and antioxidant proteins. Other examples of RSV reducing the progression of AS in the animal model are available. Studies conducted by Zhou *et al*. [113] elucidated that RSV ameliorates AS induced in high‐fat diet mice.

#### Cardiac hypertrophy

Cardiac hypertrophy (CH) is the heart's response to stress, such as pressure overload, which can develop into heart failure. Fan *et al*. [116] found that breast cancer type 1 (BRCA1) susceptibility protein inactivation can accelerate the expression of MicroRNA‐155 (miR‐155), which eventually leads to CH. The study showed that RSV partially reduces CH by down‐regulating miR‐155 expression. The cardioprotective effects of RSV may be due to its ability to retard the progression of AS by altering the gut microbiota. A second study showed that RSV increased the number of *Bifidobacterium* spp. and *Lactobacillus* [117]. The salient features of RSV make it a potent candidate in functional foods and as a cardioprotective agent.

RSV has been administered to spontaneously hypertensive rats (SHRs) with high blood pressure and concentric hypertrophy. Without reducing blood pressure, RSV therapy dramatically decreased concentric hypertrophy, and systolic and diastolic dysfunction in SHR. RSV dramatically lowered oxidative stress levels in SHR, comparable to earlier research [118]. Dong *et al*. [119] also noted that RSV effectively prevented the transition of cardiac hypertrophy, but the reduction is time‐dependent on treatment. Rats with CH were given RSV and beneficial effects were observed through protecting the cardiac structure and modulation of Ca^2+^ handling proteins. A comparative study using equal doses of RSV and its derivatives for 8 weeks improved diastolic function and exerted some cardioprotective and antihypertrophic effects. The study concluded that the acute as well as chronic protection provided by combination treatment with RSV may be due to pro‐angiogenic, anti‐hyperlipidemic and anti‐apoptotic effects [120] even though some studies are showing that RSV increases the concentration of cardiovascular biomarkers [121].

### RSV derivatives

In recent years, there have been studies to further derive new compounds for RSV, hoping to prolong the excretion of RSV *in vivo* and directly increase the intensity of physiological activity [122, 123]. Research on chemically synthesized derivatives of RSV is the most high‐profile research in this area in recent years, including RSV ester derivatives, RSV flavonoid derivatives, RSV curcumin complex derivatives and imine group RSV derivatives, etc. [124, 125, 126]; furthermore, many studies also tried to use nanocarriers to coat RSV to increase its bioavailability [127]. Chemical modifications such as esterification and ethanol dissolution have improved the solubility of RSV in foods and edible oils [128]. The oral bioavailability for RSV was 20–29.8% [111]. Chen *et al*. [125] reported that RSV can be combined with pyrrolidine to form RSV flavonoid derivatives after hydroformylation and found that RSV flavonoid derivatives had better anti‐inflammatory ability [125]. However, in addition to simple derivatization of RSV to improve the application of RSV, especially in the development of anticancer drugs, many new derivatives and analogs of RSV have been synthesized using different modification strategies to overcome these limitations and improve anticancer efficacy [129]. Xu *et al*. [130] showed that using cationic peptide liposomes to encapsulate RSV and p53 genes has strong anticancer effects on cervical and breast cancer cells. Liposomes improve drug delivery capabilities and can be used as solubilizers for low‐solubility and targeted drugs [131].

#### Research strategies for structural modification of RSV

Structure–activity relationship studies have shown that RSV has a stilbene moiety structure with anti‐inflammatory properties, and any modification in the RSV parent structure is crucial to its specific biological activity [132]. The antioxidant effects of RSV have been the subject of intense research over the past few decades. The phenolic –OH groups on the molecular structure of RSV, especially 4′‐OH and trans‐conformation, are the reason for RSV's high antioxidant effect [133, 134]. The antioxidant properties of RSV are related to the chemical function of the hydroxyl group [135], and the 4′‐OH and trans stereochemistry in its molecular structure are involved in inhibiting cell growth [136]. Compared with the previous antioxidative research on 3‐OH and 5‐OH in the RSV structure, the 4′‐OH group in the RSV structure exhibited more significant antioxidant activity [137, 138, 139].

As such, when trying to solve the bottleneck issue of the low utilization rate of RSV by chemical modification, three primary factors should be considered: (a) whether it can be modified without destroying the conjugated double bond (conjugated double bond, –C=C–C under the premise of retaining its excellent anti‐oxidation characteristics), and then carry out the modification reaction of –OH group. (b) Can the modified –OH group improve RSV's biological activity and bioavailability without cell or animal toxicity? (c) Does the derivatized modified –OH group have the potential to be biodegraded and is the released product a component that organisms can metabolize? The conjugate effect, or electron delocalization, is an essential structure in free radical scavenging ability. RSV is a typical example. Its structure not only has two electron‐withdrawing group (electron‐withdrawing group) phenol compound structures, but also has a conjugated double‐bond skeleton that connects the two phenol compound structures with continuous single/double bonds. Therefore, if the conjugated electron skeleton of the continuous single/double bond is modified, the overall electronic conjugated system of RSV will be destroyed [140].

#### Research on chemical modification of fatty acid esterification of RSV

**Fig. 2:**
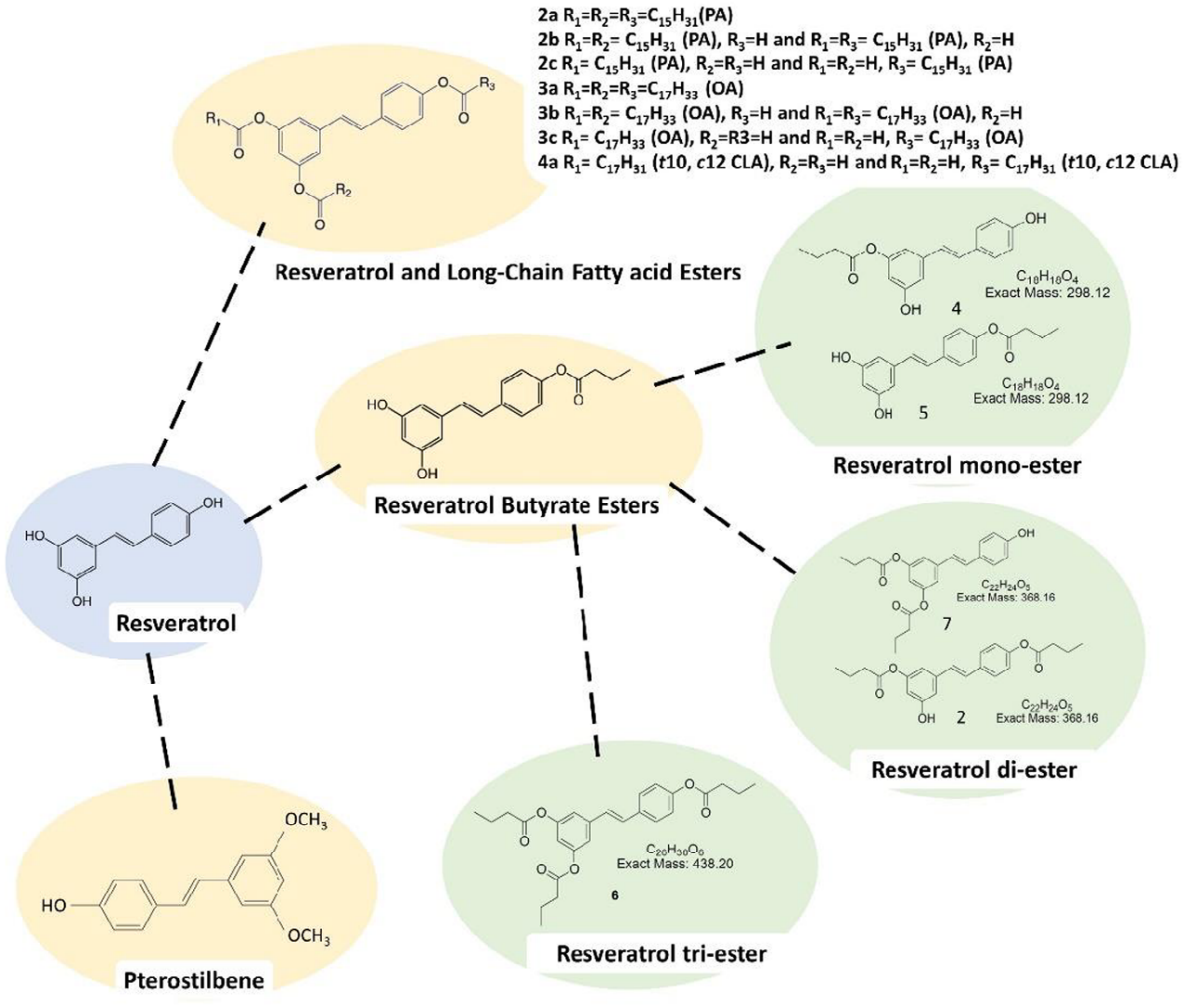
Chemical structure of RSV, RSV butyl esters and pterostilbene. Fatty acid esterifies with –OH groups (3‐OH, 5‐OH, 4'‐OH) on RSV to form fatty acid‐esterified RSV derivatives. The identified structure types are RBE mono‐esters (Nos 4 and 5), RBE di‐esters (Nos 2 and 7), and RBE tri‐esters (No. 6).

In a study by Oh *et al*. [126], fatty acids were used to esterify RSV to form RSV esters, in an attempt to prolong the residency time of RSV in the body by increasing the lipophilicity of RSV. In addition, Oh *et al*. [126] found that RSV esters showed higher antioxidant capacity in lipophilic antioxidant experiments [114]. Szczepańska *et al*. [141] performed a lyophilization process on RSV using palmitic acid (PA), oleic acid (OA) and conjugated linoleic acid (CLA) in order to expand the possible application of RSV with enhanced biological activity and increase the health benefits of long‐chain fatty acids. Cancer cell lines were treated with diesters and triesters to evaluate their anticancer and antioxidant properties (Fig. 2, RSV and long‐chain fatty acids esters). The results showed that esters of RSV and long‐chain fatty acids can enhance their biological activity. RSV derivatives have potential applications in cancer prevention, treatment, and oxidative stress suppression.

In addition, 12 different fatty acids were reacted with RSV to produce RSV derivatives with varying chain lengths and degrees of saturation (C3:0‐C22:6). Among these RSV derivatives, the chain length RSV derivatives of chain fatty acids (C18:0 and C18:1) showed higher antioxidant activity in the DPPH free radical scavenging capacity assay. In contrast, short‐chain fatty acid (C3:0, C4:0, and C6:0) links make resveratrol derivatives more effective than RSV in terms of ABTS's free radical scavenging ability [126, 142].

Our previous study selected short‐chain fatty acids (SCFAs) as candidate molecules for chemical modification; using SCFAs and three –OH groups on RSV (3‐OH, 5‐OH, 4′‐OH) we generated short‐chain fatty acid esterified RSV derivatives. As well as reporting that a short esterification reaction will not destroy the RSV conjugate characteristics. Hu *et al*. [143] also reported that RSV esters can be hydrolyzed into free RSV and fatty acids by pancreatic lipase in the *in vitro* gastrointestinal digestive system. Tain *et al*. reported that *N*‐ethyl‐*N*′‐(3 dimethylaminopropyl) carbodiimide (EDC) and 4‐dimethyl aminopyridine (DMAP) improve the Steglich esterification reaction [144] to produce short‐chain fatty acids (acetic acid, propionic acid, butyric acid) and white RSV esters; in the case of butylated RSV, yield increased by 30% [145]. Moreover, our previous study further confirmed that the esterification of RSV can increase the biological activity of RSV [122]. Since SCFAs are biochemical molecules that are ubiquitous in the intestinal environment [146], many studies have shown that RSV can adjust the intestinal flora, which may be related to the increase in the concentration of SCFAs in the intestine [147]. In Fig. 2, the chemical structures No. 4 (RSV mono‐ester) and No. 7 (RSV di‐ester) belong to the RSV esterification products that retain the 4′‐OH group.

RSV butyrate esters (RBEs) are RSV and butyric acid derivatives, having biological properties comparable to RSV but with increased bioavailability. Our previous studies reported RBEs synthesized by esterification of butyric acid and RSV, trying to improve the low bioavailability of original RSV and evaluating RBEs *in vitro* [145], in cell models [122], and animal experiments [102, 148]; antioxidant capacity, lipid biosynthesis, candidate gene regulation, liver protection ability, obesity inhibition and intestinal bacteria adjustment effects were also examined. RBEs complexes have been identified as having significant antioxidant and anti‐fat accumulation capacities. Shih *et al*. [149] reported, for the first time, the isolation, identification, and bioactive properties of RBE derivatives, which are essential for its practical application as a therapeutic agent.

#### Antioxidant activity of RBEs *in vitro*

With regard to lipid antioxidant capacity, our previous studies measured the value of conjugated diene and *p*‐anisidine in corn oil without antioxidants, performed *β*‐carotene bleaching assay and Cu^2+^−induced low‐density lipoprotein oxidation, and compared RSV and lipid antioxidant capacity of RBEs [145]. The results showed that although RBEs had the highest inhibition rate (79.0%) in Cu^2+^−induced LDL oxidation, their effects on inhibiting conjugated diene, p‐anisidine and β‐carotene were similar to RSV's. In other words, RBEs retain their ability to chemically inhibit lipid oxidation [145]. Zheng *et al*. reported that the co‐existence of RSV and Cu^2+^ resulted in the formation of Cu^2+^−RE complexes with intense oxidative activity of free radicals, which intercalated into DNA [150]. Oxidation of low‐density lipoprotein (LDL) is a risk factor for atherosclerosis and coronary heart disease, and low and high concentrations of oxidized LDL can induce inflammation and apoptosis, respectively [151]. Therefore, substituting the hydroxyl group of RSV with an ester group led to a change in the reaction site with Cu^2+^, thus preventing oxidation from proceeding. It can be seen that the –OH position substituted by butyric acid on the RBE monomer changes the original biological activity of RSV [145].

#### RBEs decrease lipid biosynthesis in cell models

Tain *et al*. [122] examined the effects of RBEs and RSV on lipid metabolism in HepG2 cells cultured in the presence of oleic acid. The results showed that compared with RSV, RBEs (12.5 μm) only needed 1/4 dose to have the same activity in reducing lipid accumulation in cells. This effect was mediated by the downregulation of p‐ACC (phosphorylation of acetyl‐CoA carboxylase) and SREBP‐2 expression and was more substantial than RSV. Previous studies have shown that RSV can not only effectively regulate Sirt‐1 and PPAR‐γ and prevent fat accumulation in 3T3‐L1 adipocytes [152], but also effectively alleviate fat accumulation and reduce the concentration of intracellular triglyceride (TG) in HepG2 cells [153], as well as activate AMPK and ACC (acetyl‐CoA carboxylase) and other energy metabolism‐related pathways, and effectively regulate the expression of SREBP‐1c and lipoproteins. The studies confirmed that RBEs are more effective than RSV at the same concentration, which may be related to the changes in the structure and lipophilicity of RBEs.

In addition, Shih *et al*. [154] reported that the mRNA levels of PPARγ, C/EBPα, FABP4, and FAS in 3T3‐L1 adipocytes treated with RSV for 10 days were significantly lower than those in the induction group. Furthermore, the mRNA levels of PPARγ, C/EBPα, FABP4, and FA were increased dramatically in 3T3‐L1 adipocytes exposed to RBEs for 10 days, respectively, compared with the untreated group. We proposed that RSV and RBEs significantly regulate lipid metabolism in 3T3‐L1 adipocytes by reducing these adipogenic regulatory molecules. Furthermore, to investigate whether activation of AMPK is related to RBE or RSV‐mediated inhibition of adipocyte differentiation, researchers examined phosphorylation of AMPK. RSV and RBEs were applied to observe the effect on lipid metabolism protein expression in 3T3‐L1 adipocytes. Compared with the control, the protein expression levels of p‐AMPK/AMPK upon RSV treatment were significantly decreased. When 3T3‐L1 adipocytes were given RBEs, the protein expression of p‐AMPK/AMPK was significantly elevated. These results suggest that RBE administration can promote AMPK activity to enhance lipid catabolism, thereby reducing lipid synthase [154].

#### Biological activity of RBEs in animal models

Perinatal exposure to BPA resulted in weight gain, lipid accumulation, elevated blood lipid levels, and deterioration of gut microbiota in female rat offspring. Shih *et al*. [102] reported that supplementation with RBEs reduced BPA‐induced weight gain and lipid accumulation, optimized blood lipid levels, significantly decreased the F/B ratio, and increased and decreased the abundance of S24‐7 and *Lactobacillus*, respectively. The results showed that supplementation with RSV and RBEs can adjust the intestinal microbiota. Further comparison of the microbiota and the physiological values of blood lipids revealed that the levels of different blood lipid biochemical molecules were associated with the growth and decline of specific bacterial species. Furthermore, analysis of fecal short‐chain fatty acid (SCFA) levels showed that BPA exposure increased fecal acetate concentrations, which could be reduced by supplementation with RBEs. However, fecal propionate and butyrate concentrations were significantly lower than acetate and did not change significantly in response to BPA exposure or RBE supplementation. Therefore, RBEs can suppress BPA‐induced obesity in female rat offspring and exhibits excellent regulatory activity on gut microbiota, which has potential application in perinatal studies [102].

Liao *et al*. [148] orally administered BPA and/or RSV/RBEs to 15‐week‐old SD pregnant female rats and evaluated male rat offspring. RBEs and RSV enhanced the expression of antioxidant‐related genes and enzyme activities in rat liver cells and inhibited oxidative damage. Furthermore, RBEs enhanced the expression of selected genes and induced extramedullary hematopoiesis and monocyte infiltration. RBEs increased the abundance of S24‐7 and Adlercreutzia in the gut of male rat offspring and the concentration of short‐chain fatty acids (SCFAs) in feces. SCFAs promote the induction and expansion of intestinal regulatory T cells [155], Dendritic cells and macrophages [156] exercise an anti‐carcinogenic and anti‐oxidative effect in the intestine [157], and inhibit pathogen‐induced inflammation [158]. RBEs also increase the antioxidant capacity of the liver by inducing Nrf2 and promoting the expression of HO‐1, SOD and CAT. It also increased the concentration of intestinal SCFAs and strengthened the barrier formed by intestinal cells, thereby preventing BPA‐induced metabolic derangement in male rat offspring, and reduced liver inflammation. This study identified mechanisms underlying the protective effect of RBEs on liver injury induced by periconceptional BPA exposure and the impact of the gut microbiota on the gut‐liver axis in rat offspring [148].

#### Separation and purification of RBE monomers

Shih *et al*. [149] successfully separated, purified and identified a mixture of RBEs via EI‐MS, IR, and nuclear magnetic resonance (NMR) spectroscopy (^1^H NMR and ^13^C NMR). The identified structure types were RBE mono‐ester (Nos 4 and 5), RBE di‐ester (Nos 2 and 7), and RBE tri‐ester (No. 6). The relevant structures, formula and codes are listed in Fig. 2. Since Nos 2 and 4 are the main ester derivatives in the RBE mixture in the obtained purified samples, the biological activity of HepG2 was investigated with these two purified products and RBEs. Antioxidant tests showed that Nos 2 and 4 of the sample (50 μm) were 1.8 and 2.9 times greater than RBEs at reducing the ability of H_2_O_2_ (200 μm) to induce ROS generation, respectively. The overall results showed that Nos 2 and 4 could successfully retain the antioxidant properties of RSV [149].

## Future perspectives

From the available reports, increasing evidence has demonstrated the vital role of the gut microbiota and TMAO in the progression of CVD. The available data indicates that TMAO can be used as a candidate marker molecule for new therapeutic strategies against CVD. Additionally, inhibition of TMAO alone may not effectively regulate the progress of cardiovascular events. Therefore, polyphenol molecules with multiple physiological activities, especially RSV and its derivatives, have great application potential as a treatment for regulating TMAO *in vivo* in the future. In addition, due to the critical differences between mice and humans, more details have to be uncovered to understand the exact mechanism in humans. Furthermore, human trials and specific factors, such as overall diet, lifestyle choices, genetic predispositions, dietary habits and other underlying health conditions, should be considered. More accurate research is required for TMAO‐targeted diagnosis of CVD and to apply RSV as a therapeutic agent against cardiovascular events.

## Conclusion

Different reports found that RSV and its derivatives, related to phenolic compounds, have diverse biological activities and nutritional properties that contribute to controlling various diseases, especially CVD, with strong therapeutic potential. The evidence is also consistent with *in vivo* studies. Since the bioavailability of RSV is significantly less, derivatization of the compound to improve its absorption and bioactivity is essential. Recent studies are showing the way toward development of efficient therapeutic strategies. However, detailed clinical studies are needed to establish causality, such as randomized controlled trials or experimental studies, to determine whether manipulating TMAO levels directly affects the occurrence of cardiovascular events. Determining the effects of RSV availability, dose, intervention time, and its derivatives on TMAO levels is necessary to establish a causal relationship between TMAO and cardiovascular disease.

## Conflict of interest

The authors declare no conflict of interest.

## Author contributions

Conceptualization, C‐YH; SHH and M‐KS; resources, Y‐WC; writing—original draft preparation, C‐YH and SHH; writing—review and editing, C‐WH, D‐QC and R‐YL; visualization, Y‐LT, Y‐WC; supervision, M‐KS; project administration, C‐YH; funding acquisition, C‐YH, M‐KS. All authors have read and agreed to the published version of the manuscript.

## References

[1] Marzullo P , Di Renzo L , Pugliese G , De Siena M , Barrea L , Muscogiuri G , Colao A , Savastano S and Obesity Programs of Nutrition Research and Assessment (OPERA) Group (2020) From obesity through gut microbiota to cardiovascular diseases: a dangerous journey. Int J Obes Suppl 10, 35–49. 32714511 10.1038/s41367-020-0017-1 PMC7371682

[2] Singh Thakur J , Nangia R and Singh S (2021) Progress and challenges in achieving noncommunicable diseases targets for the sustainable development goals. FASEB Bioadv 3, 563–568. 34377953 10.1096/fba.2020-00117 PMC8332469

[3] Guasti L , Galliazzo S , Molaro M , Visconti E , Pennella B , Gaudio GV , Lupi A , Grandi AM and Squizzato A (2021) TMAO as a biomarker of cardiovascular events: a systematic review and meta‐analysis. Intern Emerg Med 16, 201–207. 32779113 10.1007/s11739-020-02470-5

[4] Roncal C , Martínez‐Aguilar E , Orbe J , Ravassa S , Fernandez‐Montero A , Saenz‐Pipaon G , Ugarte A , Estella‐Hermoso de Mendoza A , Rodriguez JA , Fernández‐Alonso S *et al*. (2019) Trimethylamine‐N‐oxide (TMAO) predicts cardiovascular mortality in peripheral artery disease. Sci Rep 9, 15580. 31666590 10.1038/s41598-019-52082-z PMC6821861

[5] Wiedeman AM , Barr SI , Green TJ , Xu Z , Innis SM and Kitts DD (2018) Dietary choline intake: current state of knowledge across the life cycle. Nutrients 10, 1513. 30332744 10.3390/nu10101513 PMC6213596

[6] Shi W , Mersfelder J and Hille R (2005) The interaction of trimethylamine dehydrogenase and electron‐transferring Flavoprotein. J Biol Chem 280, 20239–20246. 15760891 10.1074/jbc.M500582200

[7] Zhang Y , Wang Y , Ke B and Du J (2021) TMAO: how gut microbiota contributes to heart failure. Transl Res 228, 109–125. 32841736 10.1016/j.trsl.2020.08.007

[8] Trøseid M , Andersen GØ , Broch K and Hov JR (2020) The gut microbiome in coronary artery disease and heart failure: current knowledge and future directions. EBioMedicine 52, 102649. 32062353 10.1016/j.ebiom.2020.102649 PMC7016372

[9] Jayachandran M , Chung SSM and Xu B (2020) A critical review on diet‐induced microbiota changes and cardiovascular diseases. Crit Rev Food Sci Nutr 60, 2914–2925. 31552753 10.1080/10408398.2019.1666792

[10] Annunziata G , Ciampaglia R , Maisto M , D'Avino M , Caruso D , Tenore GC , Novellino E , D'avino M , Caruso D , Tenore GC *et al*. (2021) Taurisolo®, a grape pomace polyphenol nutraceutical reducing the levels of serum biomarkers associated with atherosclerosis. Front Cardiovasc Med 8, 697272. 34350218 10.3389/fcvm.2021.697272 PMC8326362

[11] Koushki M , Amiri‐Dashatan N , Ahmadi N , Abbaszadeh HA and Rezaei‐Tavirani M (2018) Resveratrol: a miraculous natural compound for diseases treatment. Food Sci Nutr 6, 2473–2490. 30510749 10.1002/fsn3.855 PMC6261232

[12] Chen M , YiL ZY , Zhou X , Ran L , Yang J , Zhu J , Zhang Q and Mi M (2016) Resveratrol attenuates trimethylamine‐N‐oxide (TMAO)‐induced atherosclerosis by regulating TMAO synthesis and bile acid metabolism via remodeling of the gut microbiota. MBio 7, e02210‐15. 27048804 10.1128/mBio.02210-15 PMC4817264

[13] Landfald B , Valeur J , Berstad A and Raa J (2017) Microbial trimethylamine‐N‐oxide as a disease marker: something fishy? Microb Ecol Health Dis 28, 1327309. 28588431 10.1080/16512235.2017.1327309 PMC5444358

[14] Lidbury I , Murrell JC and Chen Y (2014) Trimethylamine N‐oxide metabolism by abundant marine heterotrophic bacteria. Proc Natl Acad Sci USA 111, 2710–2715. 24550299 10.1073/pnas.1317834111 PMC3932874

[15] Velasquez MT , Ramezani A , Manal A and Raj DS (2016) Trimethylamine N‐oxide: the good, the bad and the unknown. Toxins (Basel) 8, 326. 27834801 10.3390/toxins8110326 PMC5127123

[16] Janeiro MH , Ramírez MJ , Milagro FI , Martínez JA and Solas M (2018) Implication of trimethylamine N‐oxide (TMAO) in disease: potential biomarker or new therapeutic target. Nutrients 10, 1398. 30275434 10.3390/nu10101398 PMC6213249

[17] Schmidt AC and Leroux J‐C (2020) Treatments of trimethylaminuria: where we are and where we might be heading. Drug Discov Today 25, 1710–1717. 32615074 10.1016/j.drudis.2020.06.026

[18] Stubbs JR , House JA , Ocque AJ , Zhang S , Johnson C , Kimber C , Schmidt K , Gupta A , Wetmore JB , Nolin TD *et al*. (2016) Serum trimethylamine‐N‐oxide is elevated in CKD and correlates with coronary atherosclerosis burden. J Am Soc Nephrol 27, 305–313. 26229137 10.1681/ASN.2014111063 PMC4696571

[19] Zhu W , Gregory JC , Org E , Buffa JA , Gupta N , Wang Z , Li L , Fu X , Wu Y , Mehrabian M *et al*. (2016) Gut microbial metabolite TMAO enhances platelet Hyperreactivity and thrombosis risk. Cell 165, 111–124. 26972052 10.1016/j.cell.2016.02.011 PMC4862743

[20] Ding L , Chang M , Guo Y , Zhang L , Xue C , Yanagita T , Zhang T and Wang Y (2018) Trimethylamine‐N‐oxide (TMAO)‐induced atherosclerosis is associated with bile acid metabolism. Lipids Health Dis 17, 286. 30567573 10.1186/s12944-018-0939-6 PMC6300890

[21] Liu Y and Dai M (2020) Trimethylamine N‐oxide generated by the gut microbiota is associated with vascular inflammation: new insights into atherosclerosis. Mediators Inflamm 2020, 4634172. 32148438 10.1155/2020/4634172 PMC7048942

[22] Gawrys‐Kopczynska M , Konop M , Maksymiuk K , Kraszewska K , Derzsi L , Sozanski K , Holyst R , Pilz M , Samborowska E , Dobrowolski L *et al*. (2020) TMAO, a seafood‐derived molecule, produces diuresis and reduces mortality in heart failure rats. Elife 9, e57028. 32510330 10.7554/eLife.57028 PMC7334024

[23] Kelly RH and Yancey PH (1999) High contents of trimethylamine oxide correlating with depth in deep‐sea teleost fishes, skates, and decapod crustaceans. Biol Bull 196, 18–25. 25575382 10.2307/1543162

[24] Yang S , LiXX YF , Zhao R , Pan X , Liang J , Tian L , Li XX , Liu L , Xing Y and Wu M (2019) Gut microbiota‐dependent marker TMAO in promoting cardiovascular disease: inflammation mechanism, clinical prognostic, and potential as a therapeutic target. Front Pharmacol 10, 1360. 31803054 10.3389/fphar.2019.01360 PMC6877687

[25] Wang Z , Bergeron N , Levison BS , Li XS , Chiu S , Jia X , Koeth RA , Li L , Wu Y , Tang WHW *et al*. (2019) Impact of chronic dietary red meat, white meat, or non‐meat protein on trimethylamine N‐oxide metabolism and renal excretion in healthy men and women. Eur Heart J 40, 583–594. 30535398 10.1093/eurheartj/ehy799 PMC6374688

[26] Bernstein AM , Sun Q , Hu FB , Stampfer MJ , Manson JE and Willett WC (2010) Major dietary protein sources and risk of coronary heart disease in women. Circulation 122, 876–883. 20713902 10.1161/CIRCULATIONAHA.109.915165 PMC2946797

[27] Ufnal M and Nowiński A (2019) Is increased plasma TMAO a compensatory response to hydrostatic and osmotic stress in cardiovascular diseases? Med Hypotheses 130, 109271. 31383335 10.1016/j.mehy.2019.109271

[28] Cho CE , Taesuwan S , Malysheva OV , Bender E , Tulchinsky NF , Yan J , Sutter JL and Caudill MA (2017) Trimethylamine‐N‐oxide (TMAO) response to animal source foods varies among healthy young men and is influenced by their gut microbiota composition: a randomized controlled trial. Mol Nutr Food Res 61, doi: 10.1002/mnfr.201600324 27377678

[29] Aadland EK , Lavigne C , Graff IE , Eng Ø , Paquette M , Holthe A , Mellgren G , Jacques H and Liaset B (2015) Lean‐seafood intake reduces cardiovascular lipid risk factors in healthy subjects: results from a randomized controlled trial with a crossover design. Am J Clin Nutr 102, 582–592. 26224298 10.3945/ajcn.115.112086

[30] Coutinho‐Wolino KS , Cardozo LFMF , de Oliveira Leal V , Mafra D and Stockler‐Pinto MB (2021) Can diet modulate trimethylamine N‐oxide (TMAO) production? What do we know so far? Eur J Nutr 60, 3567–3584. 33533968 10.1007/s00394-021-02491-6

[31] Novakovic M , Rout A , Kingsley T , Kirchoff R , Singh A , Verma V , Kant R and Chaudhary R (2020) Role of gut microbiota in cardiovascular diseases. World J Cardiol 12, 110–122. 32431782 10.4330/wjc.v12.i4.110 PMC7215967

[32] Koeth RA , Wang Z , Levison BS , Buffa JA , Org E , Sheehy BT , Britt EB , Fu X , Wu Y , Li L *et al*. (2013) Intestinal microbiota metabolism of l‐carnitine, a nutrient in red meat, promotes atherosclerosis. Nat Med 19, 576–585. 23563705 10.1038/nm.3145 PMC3650111

[33] Romano KA , Vivas EI , Amador‐Noguez D and Rey FE (2015) Intestinal microbiota composition modulates choline bioavailability from diet and accumulation of the proatherogenic metabolite trimethylamine‐N‐oxide. MBio 6, e02481. 25784704 10.1128/mBio.02481-14 PMC4453578

[34] Massmig M , Reijerse E , Krausze J , Laurich C , Lubitz W , Jahn D and Moser J (2020) Carnitine metabolism in the human gut: characterization of the two‐component carnitine monooxygenase CntAB from *Acinetobacter baumannii* . J Biol Chem 295, 13065–13078. 32694223 10.1074/jbc.RA120.014266 PMC7489909

[35] Martínez‐del Campo A , Bodea S , Hamer HA , Marks JA , Haiser HJ , Turnbaugh PJ and Balskus EP (2015) Characterization and detection of a widely distributed gene cluster that predicts anaerobic choline utilization by human gut bacteria. MBio 6, e00042‐15. 25873372 10.1128/mBio.00042-15 PMC4453576

[36] Zeisel SH and Warrier M (2017) Trimethylamine N‐oxide, the microbiome, and heart and kidney disease. Annu Rev Nutr 37, 157–181. 28715991 10.1146/annurev-nutr-071816-064732

[37] Yoshida N , Yamashita T and Hirata K‐I (2018) Gut microbiome and cardiovascular diseases. Diseases 6, 56. 29966270 10.3390/diseases6030056 PMC6164700

[38] Yoo W , Zieba JK , Foegeding NJ , Torres TP , Shelton CD , Shealy NG , Byndloss AJ , Cevallos SA , Gertz E , Tiffany CR *et al*. (2021) High‐fat diet‐induced colonocyte dysfunction escalates microbiota‐derived trimethylamine N‐oxide. Science 373, 813–818. 34385401 10.1126/science.aba3683 PMC8506909

[39] Thøgersen R , Rasmussen MK , Sundekilde UK , Goethals SA , Van Hecke T , Vossen E , De Smet S and Bertram HC (2020) Background diet influences TMAO concentrations associated with red meat intake without influencing apparent hepatic TMAO‐related activity in a porcine model. Metabolites 10, 57. 32041174 10.3390/metabo10020057 PMC7074160

[40] Haro C , Rangel‐Zúñiga OA , Alcalá‐Díaz JF , Gómez‐Delgado F , Pérez‐Martínez P , Delgado‐Lista J , Quintana‐Navarro GM , Landa BB , Navas‐Cortés JA , Tena‐Sempere M *et al*. (2016) Intestinal microbiota is influenced by gender and body mass index. PLoS ONE 11, e0154090. 27228093 10.1371/journal.pone.0154090 PMC4881937

[41] Dehghan P , Farhangi MA , Nikniaz L , Nikniaz Z and Asghari‐Jafarabadi M (2020) Gut microbiota‐derived metabolite trimethylamine N‐Oxide (TMAO) potentially increases the risk of obesity in adults: an exploratory systematic review and dose‐response meta‐analysis. Obes Rev 21, e12993. 32017391 10.1111/obr.12993

[42] Friemann W , Overhoff W and Wolter JR (1959) Eye diseases in the fishing industry. Arch Gewerbepathol Gewerbehyg 17, 1–56. 13628053

[43] Fluhr JW , Kelterer D , Fuchs S , Kaatz M , Grieshaber R , Kleesz P and Elsner P (2005) Additive impairment of the barrier function and irritation by biogenic amines and sodium lauryl Sulphate: a controlled in vivo tandem irritation study. Skin Pharmacol Physiol 18, 88–97. 15767770 10.1159/000083709

[44] Guest I and Varma DR (2011) Selective growth inhibition of the male progeny of mice treated with trimethylamine during pregnancy. Can J Physiol Pharmacol 71, 185–187. 10.1139/y93-026 8319141

[45] Jaworska K , Konop M , Hutsch T , Perlejewski K , Radkowski M , Grochowska M , Bielak‐Zmijewska A , Mosieniak G , Sikora E and Ufnal M (2020) Trimethylamine but not trimethylamine oxide increases with age in rat plasma and affects smooth muscle cells viability. J Gerontol A Biol Sci Med Sci 75, 1276–1283. 31411319 10.1093/gerona/glz181

[46] Restini CB , Fink GD and Watts SW (2018) Abstract P 145: the bacterial metabolite trimethylamine (TMA), but not trimethylamine N‐oxide (TMAO), causes vascular contraction. Hypertension 72, doi: 10.1161/hyp.72.suppl_1.P145

[47] Jaworska K , Hering D , Mosieniak G , Bielak‐Zmijewska A , Pilz M , Konwerski M , Gasecka A , Kaplon‐Cieślicka A , Filipiak K , Sikora E *et al*. (2019) TMA, A forgotten uremic toxin, but not TMAO, is involved in cardiovascular pathology. Toxins (Basel) 11, 490. 31454905 10.3390/toxins11090490 PMC6784008

[48] Bennett BJ , Vallim TQA , Wang Z , Shih DM , Meng Y , Gregory J , Allayee H , Lee R , Graham M , Crooke R *et al*. (2013) Trimethylamine‐N‐oxide, a metabolite associated with atherosclerosis, exhibits complex genetic and dietary regulation. Cell Metab 17, 49–60. 23312283 10.1016/j.cmet.2012.12.011 PMC3771112

[49] Videja M , Vilskersts R , Korzh S , Cirule H , Sevostjanovs E , Dambrova M and Makrecka‐Kuka M (2021) Microbiota‐derived metabolite trimethylamine N‐oxide protects mitochondrial energy metabolism and cardiac functionality in a rat model of right ventricle heart failure. Front Cell Dev Biol 8, 622741. 33520996 10.3389/fcell.2020.622741 PMC7841203

[50] Wilson A , McLean C and Kim RB (2016) Trimethylamine‐N‐oxide: a link between the gut microbiome, bile acid metabolism, and atherosclerosis. Curr Opin Lipidol 27, 148–154. 26959704 10.1097/MOL.0000000000000274

[51] Jang HR and Lee H‐Y (2021) Mechanisms linking gut microbial metabolites to insulin resistance. World J Diabetes 12, 730–744. 34168724 10.4239/wjd.v12.i6.730 PMC8192250

[52] Heianza Y , Ma W , Manson JAE , Rexrode KM and Qi L (2017) Gut microbiota metabolites and risk of Major adverse cardiovascular disease events and death: a systematic review and meta‐analysis of prospective studies. J Am Heart Assoc 6, e004947. 28663251 10.1161/JAHA.116.004947 PMC5586261

[53] Qi J , You T , Li J , Pan T , Xiang L , Han Y and Zhu L (2018) Circulating trimethylamine N‐oxide and the risk of cardiovascular diseases: a systematic review and meta‐analysis of 11 prospective cohort studies. J Cell Mol Med 22, 185–194. 28782886 10.1111/jcmm.13307 PMC5742728

[54] Mueller DM , Allenspach M , Othman A , Saely CH , Muendlein A , Vonbank A , Drexel H and von Eckardstein A (2015) Plasma levels of trimethylamine‐N‐oxide are confounded by impaired kidney function and poor metabolic control. Atherosclerosis 243, 638–644. 26554714 10.1016/j.atherosclerosis.2015.10.091

[55] Peter S , Chopra S and Jacob JJ (2013) A fish a day, keeps the cardiologist away! – a review of the effect of omega‐3 fatty acids in the cardiovascular system. Indian J Endocrinol Metab 17, 422–429. 23869297 10.4103/2230-8210.111630 PMC3712371

[56] Awuchi CG , Chukwu CN , Iyiola AO , Noreen S , Morya S , Adeleye AO , Twinomuhwezi H , Leicht K , Mitaki NB and Okpala COR (2022) Bioactive compounds and therapeutics from fish: revisiting their suitability in functional foods to enhance human wellbeing. Biomed Res Int 2022, 3661866. 36033572 10.1155/2022/3661866 PMC9410824

[57] Raatz SK , Silverstein JT , Jahns L and Picklo MJ (2013) Issues of fish consumption for cardiovascular disease risk reduction. Nutrients 5, 1081–1097. 23538940 10.3390/nu5041081 PMC3705336

[58] Yu D , Shu XO , Rivera ES , Zhang X , Cai Q , Calcutt MW , Xiang YB , Li H , Gao YT , Wang TJ *et al*. (2019) Urinary levels of trimethylamine‐N‐oxide and incident coronary heart disease: a prospective investigation among urban Chinese adults. J Am Heart Assoc 8, e010606. 30606084 10.1161/JAHA.118.010606 PMC6405718

[59] Wang Z , Klipfell E , Bennett BJ , Koeth R , Levison BS , Dugar B , Feldstein AE , Britt EB , Fu X , Chung Y‐MM *et al*. (2011) Gut flora metabolism of phosphatidylcholine promotes cardiovascular disease. Nature 472, 57–63. 21475195 10.1038/nature09922 PMC3086762

[60] Zhu W , Wang Z , Tang WHW and Hazen SL (2017) Gut microbe‐generated trimethylamine N‐oxide from dietary choline is Prothrombotic in subjects. Circulation 135, 1671–1673. 28438808 10.1161/CIRCULATIONAHA.116.025338 PMC5460631

[61] Simó C and García‐Cañas V (2020) Dietary bioactive ingredients to modulate the gut microbiota‐derived metabolite TMAO. New opportunities for functional food development. Food Funct 11, 6745–6776. 32686802 10.1039/d0fo01237h

[62] Liu S , He F , Zheng T , Wan S , Chen J , Yang F , Xu X and Pei X (2021) Ligustrum robustum alleviates atherosclerosis by decreasing serum TMAO, modulating gut microbiota, and decreasing bile acid and cholesterol absorption in mice. Mol Nutr Food Res 65, 2100014. 10.1002/mnfr.202100014 34005835

[63] Li T , Tan Y , Ouyang S , He J and Liu L (2022) Resveratrol protects against myocardial ischemia‐reperfusion injury via attenuating ferroptosis. Gene 808, 145968. 34530090 10.1016/j.gene.2021.145968

[64] Wang P , Ma Y , Wang D , Zhao W , Hu X , Chen F and Zhao X (2022) Protective effects of dietary resveratrol against chronic low‐grade inflammation mediated through the gut microbiota in high‐fat diet mice. Nutrients 14, 1994. 35631150 10.3390/nu14101994 PMC9143590

[65] Annunziata G , Maisto M , Schisano C , Ciampaglia R , Narciso V , Tenore GC and Novellino E (2019) Effects of grape pomace polyphenolic extract (Taurisolo®) in reducing tmao serum levels in humans: preliminary results from a randomized, placebo‐controlled, cross‐over study. Nutrients 11, 139. 30634687 10.3390/nu11010139 PMC6356416

[66] Liao W , Yin X , Li Q , Zhang H , Liu Z , Zheng X , Zheng L and Feng X (2018) Resveratrol‐induced white adipose tissue browning in obese mice by remodeling fecal microbiota. Molecules 23, 3356. 30567366 10.3390/molecules23123356 PMC6321286

[67] Cottart C‐HCH , Nivet‐Antoine V , Laguillier‐Morizot C and Beaudeux J‐LJL (2010) Resveratrol bioavailability and toxicity in humans. Mol Nutr Food Res 54, 7–16. 20013887 10.1002/mnfr.200900437

[68] Cottart CH , Nivet‐Antoine V and Beaudeux JL (2014) Review of recent data on the metabolism, biological effects, and toxicity of resveratrol in humans. Mol Nutr Food Res 58, 7–21. 23740855 10.1002/mnfr.201200589

[69] Patel KR , Scott E , Brown VA , Gescher AJ , Steward WP and Brown K (2011) Clinical trials of resveratrol. Ann N Y Acad Sci 1215, 161–169. 21261655 10.1111/j.1749-6632.2010.05853.x

[70] Raj P , Thandapilly SJ , Wigle J , Zieroth S and Netticadan T (2021) A comprehensive analysis of the efficacy of resveratrol in atherosclerotic cardiovascular disease, myocardial infarction and heart failure. Molecules 26, 6600. 34771008 10.3390/molecules26216600 PMC8587649

[71] Golovinskaia O and Wang CK (2021) Review of functional and pharmacological activities of berries. Molecules 26, 3904. 34202412 10.3390/molecules26133904 PMC8271923

[72] Iglesias‐Carres L , Hughes MD , Steele CN , Ponder MA , Davy KP and Neilson AP (2021) Use of dietary phytochemicals for inhibition of trimethylamine N‐oxide formation. J Nutr Biochem 91, 108600. 33577949 10.1016/j.jnutbio.2021.108600

[73] Annunziata G , Maisto M , Schisano C , Ciampaglia R , Narciso V , Hassan STS , Tenore GC and Novellino E (2019) Effect of grape pomace polyphenols with or without pectin on TMAO serum levels assessed by LC/MS‐based assay: a preliminary clinical study on overweight/obese subjects. Front Pharmacol 10, 575. 31164827 10.3389/fphar.2019.00575 PMC6536651

[74] Del Rio D , Rodriguez‐Mateos A , Spencer JPE , Tognolini M , Borges G and Crozier A (2013) Dietary (poly)phenolics in human health: structures, bioavailability, and evidence of protective effects against chronic diseases. Antioxid Redox Signal 18, 1818–1892. 22794138 10.1089/ars.2012.4581 PMC3619154

[75] Suh HW , Kim SH , Park SJ , Hyun SH , Lee SY , Auh JH , Lee HJ , Cho SM , Kim JH and Choi HK (2013) Effect of Korean black raspberry (*Rubus coreanus* Miquel) fruit administration on DNA damage levels in smokers and screening biomarker investigation using 1H‐NMR‐based metabolic profiling. Food Res Int 54, 1255–1262.

[76] Liu S , You L , Zhao Y and Chang X (2018) Wild *Lonicera caerulea* berry polyphenol extract reduces cholesterol accumulation and enhances antioxidant capacity in vitro and in vivo. Food Res Int 107, 73–83. 29580541 10.1016/j.foodres.2018.02.016

[77] Chen P‐Y , Li S , Koh Y‐C , Wu J‐C , Yang M‐J , Ho C‐T and Pan M‐H (2019) Oolong tea extract and citrus Peel Polymethoxyflavones reduce transformation of l‐carnitine to trimethylamine‐N‐oxide and decrease vascular inflammation in l‐carnitine feeding mice. J Agric Food Chem 67, 7869–7879. 31287296 10.1021/acs.jafc.9b03092

[78] Bresciani L , Dall'asta M , Favari C , Calani L , Del Rio D and Brighenti F (2018) An in vitro exploratory study of dietary strategies based on polyphenol‐rich beverages, fruit juices and oils to control trimethylamine production in the colon. Food Funct 9, 6470–6483. 30465688 10.1039/c8fo01778f

[79] Li H , Sureda A , Devkota HP , Pittalà V , Barreca D , Silva AS , Tewari D , Xu S and Nabavi SM (2020) Curcumin, the golden spice in treating cardiovascular diseases. Biotechnol Adv 38, 107343. 30716389 10.1016/j.biotechadv.2019.01.010

[80] Joshi H , Bhandari U and Panda BP (2017) To assess the potential of curcumin against gut microbiota‐induced ALTERATION in choline metabolism in C57BL/6J mice. Int J Pharm Pharm Sci 9, 215–226.

[81] Shi Y , Hu J , Geng J , Hu T , Wang B , Yan W , Jiang Y , Li J and Liu S (2018) Berberine treatment reduces atherosclerosis by mediating gut microbiota in apo E^−/−^ mice. Biomed Pharmacother 107, 1556–1563. 30257374 10.1016/j.biopha.2018.08.148

[82] Zhu L , Zhang D , Zhu H , Zhu J , Weng S , Dong L , Liu T , Hu Y and Shen X (2018) Berberine treatment increases Akkermansia in the gut and improves high‐fat diet‐induced atherosclerosis in Apoe^−/−^ mice. Atherosclerosis 268, 117–126. 29202334 10.1016/j.atherosclerosis.2017.11.023

[83] Anwar S , Bhandari U , Panda BP , Dubey K , Khan W and Ahmad S (2018) Trigonelline inhibits intestinal microbial metabolism of choline and its associated cardiovascular risk. J Pharm Biomed Anal 159, 100–112. 29980011 10.1016/j.jpba.2018.06.027

[84] Cashman JR , Xiong Y , Lin J , Verhagen H , Van Poppel G , Van Bladeren PJ , Larsen‐Su S and Williams DE (1999) In vitro and in vivo inhibition of human flavin‐containing monooxygenase form 3 (FMO3) in the presence of dietary indoles. Biochem Pharmacol 58, 1047–1055. 10509757 10.1016/s0006-2952(99)00166-5

[85] Butler EJ and Fenwick GR (2019) Trimethylamine and fishy taint in eggs. World's Poult Sci J 40, 38–51.

[86] Chan JYY , Yuen ACY , Chan RYK and Chan SW (2013) A review of the cardiovascular benefits and antioxidant properties of Allicin. Phyther Res 27, 637–646. 10.1002/ptr.4796 22888009

[87] Wu WK , Panyod S , Ho CT , Kuo CH , Wu MS and Sheen LY (2015) Dietary allicin reduces transformation of L‐carnitine to TMAO through impact on gut microbiota. J Funct Foods 15, 408–417.

[88] Wu T , Gao Y , Hao J , Yin J , Li W , Geng J , Liu R , Sui W and Zhang M (2019) Lycopene, amaranth, and sorghum red pigments counteract obesity and modulate the gut microbiota in high‐fat diet fed C57BL/6 mice. J Funct Foods 60, 103437.

[89] Ryan PM , London LEE , Bjorndahl TC , Mandal R , Murphy K , Fitzgerald GF , Shanahan F , Ross RP , Wishart DS , Caplice NM *et al*. (2017) Microbiome and metabolome modifying effects of several cardiovascular disease interventions in apo‐E^−/−^ mice. Microbiome 5, 1–13. 28285599 10.1186/s40168-017-0246-x PMC5346842

[90] Pandey KB and Rizvi SI (2009) Plant polyphenols as dietary antioxidants in human health and disease. Oxid Med Cell Longev 2, 270–278. 20716914 10.4161/oxim.2.5.9498 PMC2835915

[91] Cory H , Passarelli S , Szeto J , Tamez M and Mattei J (2018) The role of polyphenols in human health and food systems: a mini‐review. Front Nutr 5, 87. 30298133 10.3389/fnut.2018.00087 PMC6160559

[92] Mehmood A , Usman M , Patil P , Zhao L and Wang C (2020) A review on management of cardiovascular diseases by olive polyphenols. Food Sci Nutr 8, 4639–4655. 32994927 10.1002/fsn3.1668 PMC7500788

[93] Xing L , Zhang H , Qi R , Tsao R and Mine Y (2019) Recent advances in the understanding of the health benefits and molecular mechanisms associated with Green tea polyphenols. J Agric Food Chem 67, 1029–1043. 30653316 10.1021/acs.jafc.8b06146

[94] Chen X , Pan S , Li F , Xu X and Xing H (2022) Plant‐derived bioactive compounds and potential health benefits: involvement of the gut microbiota and its metabolic activity. Biomolecules 12, 1871. 36551299 10.3390/biom12121871 PMC9775189

[95] Qiao Y , Sun J , Xia S , Tang X , Shi Y and Le G (2014) Effects of resveratrol on gut microbiota and fat storage in a mouse model with high‐fat‐induced obesity. Food Funct 5, 1241–1249. 24722352 10.1039/c3fo60630a

[96] Zhao L , Zhang Q , Ma W , Tian F , Shen H and Zhou M (2017) A combination of quercetin and resveratrol reduces obesity in high‐fat diet‐fed rats by modulation of gut microbiota. Food Funct 8, 4644–4656. 29152632 10.1039/c7fo01383c

[97] Jung M‐J , Lee J , Shin N‐R , Kim M‐S , Hyun D‐W , Yun J‐H , Kim PS , Whon TW and Bae J‐W (2016) Chronic repression of mTOR complex 2 induces changes in the gut microbiota of diet‐induced obese mice. Sci Rep 6, 30887. 27471110 10.1038/srep30887 PMC4965768

[98] Wilkins AT and Reimer RA (2021) Obesity, early life gut microbiota, and antibiotics. Microorganisms 9, 1–20. 10.3390/microorganisms9020413 PMC7922584 33671180

[99] Koliada A , Syzenko G , Moseiko V , Budovska L , Puchkov K , Perederiy V , Gavalko Y , Dorofeyev A , Romanenko M , Tkach S *et al*. (2017) Association between body mass index and firmicutes/bacteroidetes ratio in an adult Ukrainian population. BMC Microbiol 17, 120. 28532414 10.1186/s12866-017-1027-1 PMC5440985

[100] Koh Y‐CC , Li S , Chen P‐YY , Wu J‐CC , Kalyanam N , Ho C‐TT and Pan M‐HH (2019) Prevention of vascular inflammation by Pterostilbene via trimethylamine‐N‐oxide reduction and mechanism of microbiota regulation. Mol Nutr Food Res 63, 1900514. 10.1002/mnfr.201900514 31368236

[101] Etxeberria U , Arias N , Boqué N , Macarulla MT , Portillo MP , Martínez JA and Milagro FI (2015) Reshaping faecal gut microbiota composition by the intake of trans‐resveratrol and quercetin in high‐fat sucrose diet‐fed rats. J Nutr Biochem 26, 651–660. 25762527 10.1016/j.jnutbio.2015.01.002

[102] Shih M‐K , Tain Y‐L , Chen Y‐W , Hsu W‐H , Yeh Y‐T , Chang SKC , Liao J‐X and Hou C‐Y (2021) Resveratrol butyrate esters inhibit obesity caused by perinatal exposure to bisphenol a in female offspring rats. Molecules 26, 4010. 34209270 10.3390/molecules26134010 PMC8271435

[103] Fogacci F , Tocci G , Presta V , Fratter A , Borghi C and Cicero AFG (2019) Effect of resveratrol on blood pressure: a systematic review and meta‐analysis of randomized, controlled, clinical trials. Crit Rev Food Sci Nutr 59, 1605–1618. 29359958 10.1080/10408398.2017.1422480

[104] Liu Y , Ma W , Zhang P , He S and Huang D (2015) Effect of resveratrol on blood pressure: a meta‐analysis of randomized controlled trials. Clin Nutr 34, 27–34. 24731650 10.1016/j.clnu.2014.03.009

[105] Aydın G (2020) The effects of resveratrol on human health. Demiroğlu Bilim Univ Florence Nightingale J Med 5, 193–201.

[106] Zbikowska HM , Olas B , Wachowicz B and Krajewski T (1999) Response of blood platelets to resveratrol. Platelets 10, 247–252. 16801100 10.1080/09537109976103

[107] Lannan KL , Refaai MA , Ture SK , Morrell CN , Blumberg N , Phipps RP and Spinelli SL (2016) Resveratrol preserves the function of human platelets stored for transfusion. Br J Haematol 172, 794–806. 26683619 10.1111/bjh.13862 PMC4764392

[108] Marumo M , Ekawa K and Wakabayashi I (2020) Resveratrol inhibits Ca2+ signals and aggregation of platelets. Environ Health Prev Med 25, 70. 33160329 10.1186/s12199-020-00905-1 PMC7648989

[109] Smolenski A (2012) Novel roles of cAMP/cGMP‐dependent signaling in platelets. J Thromb Haemost 10, 167–176. 22136590 10.1111/j.1538-7836.2011.04576.x

[110] Chong MF‐F , Macdonald R and Lovegrove JA (2010) Fruit polyphenols and CVD risk: a review of human intervention studies. Br J Nutr 104, S28–S39. 20955648 10.1017/S0007114510003922

[111] Akinwumi BC , Bordun K‐AM and Anderson HD (2018) Biological activities of stilbenoids. Int J Mol Sci 19, 792. 29522491 10.3390/ijms19030792 PMC5877653

[112] Yang L , Zhang Y , Zhu M , Zhang Q , Wang X , Wang Y , Zhang J , Li J , Yang L , Liu J *et al*. (2016) Resveratrol attenuates myocardial ischemia/reperfusion injury through up‐regulation of vascular endothelial growth factor B. Free Radic Biol Med 101, 1–9. 27667182 10.1016/j.freeradbiomed.2016.09.016

[113] Zhou L , Long J , Sun Y , Chen W , Qiu R and Yuan D (2020) Resveratrol ameliorates atherosclerosis induced by high‐fat diet and LPS in Apo E^−/−^ mice and inhibits the activation of CD4+ T cells. Nutr Metab (Lond) 17, 41. 32508962 10.1186/s12986-020-00461-z PMC7251691

[114] Xu L , Wang R , Liu H , Wang J , Mang J and Xu Z (2020) Resveratrol treatment is associated with lipid regulation and inhibition of lipoprotein‐associated phospholipase A2 (Lp‐PLA2) in rabbits fed a high‐fat diet. Evid Based Complement Alternat Med 2020, 9641582. 32595754 10.1155/2020/9641582 PMC7256704

[115] Sharifi‐Rad J , Quispe C , Alfred MA , Anil Kumar NV , Lombardi N , Cinquanta L , Iriti M , Varoni EM , Gupta G , Chellappan DK *et al*. (2021) Current trends on resveratrol bioactivities to treat periodontitis. Food Biosci 42, 101205.

[116] Fan Y , Liu L , Fang K , Huang T , Wan L , Liu Y , Zhang S , Yan D , Li G , Gao Y *et al*. (2022) Resveratrol ameliorates cardiac hypertrophy by Down‐regulation of mi R‐155 through activation of breast cancer type 1 susceptibility protein. J Am Heart Assoc 5, e002648. 10.1161/JAHA.115.002648 PMC4843545 27107135

[117] Cardona F , Andrés‐Lacueva C , Tulipani S , Tinahones FJ and Queipo‐Ortuño MI (2013) Benefits of polyphenols on gut microbiota and implications in human health. J Nutr Biochem 24, 1415–1422. 23849454 10.1016/j.jnutbio.2013.05.001

[118] Thandapilly SJ , Wojciechowski P , Behbahani J , Louis XL , Yu L , Juric D , Kopilas MA , Anderson HD and Netticadan T (2010) Resveratrol prevents the development of pathological cardiac hypertrophy and contractile dysfunction in the SHR without lowering blood pressure. Am J Hypertens 23, 192–196. 19942861 10.1038/ajh.2009.228

[119] Dong Q , Wu Z , Li X , Yan J , Zhao L , Yang C , Lu J , Deng J and Chen M (2014) Resveratrol ameliorates cardiac dysfunction induced by pressure overload in rats via structural protection and modulation of Ca2+ cycling proteins. J Transl Med 12, 323. 25425099 10.1186/s12967-014-0323-x PMC4278670

[120] Penumathsa SV , Thirunavukkarasu M , Koneru S , Juhasz B , Zhan L , Pant R , Menon VP , Otani H and Maulik N (2007) Statin and resveratrol in combination induces cardioprotection against myocardial infarction in hypercholesterolemic rat. J Mol Cell Cardiol 42, 508–516. 17188708 10.1016/j.yjmcc.2006.10.018 PMC1857339

[121] Mankowski RT , You L , Buford TW , Leeuwenburgh C , Manini TM , Schneider S , Qiu P and Anton SD (2020) Higher dose of resveratrol elevated cardiovascular disease risk biomarker levels in overweight older adults – a pilot study. Exp Gerontol 131, 110821. 31891746 10.1016/j.exger.2019.110821 PMC8168448

[122] Tain Y‐L , Jheng L‐C , Chang SKC , Chen Y‐W , Huang L‐T , Liao J‐X and Hou C‐Y (2020) Synthesis and characterization of novel resveratrol butyrate esters that have the ability to prevent fat accumulation in a liver cell culture model. Molecules 25, 4199. 32937766 10.3390/molecules25184199 PMC7571132

[123] Peterson JA , Crowther CM , Andrus MB and Kenealey JD (2019) Resveratrol derivatives increase cytosolic calcium by inhibiting plasma membrane ATPase and inducing calcium release from the endoplasmic reticulum in prostate cancer cells. Biochem Biophys Rep 19, 100667. 31463373 10.1016/j.bbrep.2019.100667 PMC6709415

[124] de Freitas SM , Coelho LF , Guirelli IM , Pereira RM , Ferreira‐Silva GÁ , Graravelli GY , Horvath RO , Caixeta ES , Ionta M and Viegas C (2018) Synthetic resveratrol‐curcumin hybrid derivative inhibits mitosis progression in estrogen positive MCF‐7 breast cancer cells. Toxicol In Vitro 50, 75–85. 29501629 10.1016/j.tiv.2018.02.020

[125] Chen LZ , Yao L , Jiao MM , Shi JB , Tan Y , Ruan BF and Liu XH (2019) Novel resveratrol‐based flavonol derivatives: synthesis and anti‐inflammatory activity in vitro and in vivo. Eur J Med Chem 175, 114–128. 31077997 10.1016/j.ejmech.2019.05.004

[126] Oh WY and Shahidi F (2018) Antioxidant activity of resveratrol ester derivatives in food and biological model systems. Food Chem 261, 267–273. 29739593 10.1016/j.foodchem.2018.03.085

[127] Santos AC , Pereira I , Pereira‐Silva M , Ferreira L , Caldas M , Magalhães M , Figueiras A , Ribeiro AJ and Veiga F (2019) Nanocarriers for resveratrol delivery: impact on stability and solubility concerns. Trends Food Sci Technol 91, doi: 10.1016/j.tifs.2019.07.048

[128] Li T , Guo Q , Qu Y , Li Y , Liu H , Liu L , Zhang Y , Jiang Y and Wang Q (2022) Solubility and physicochemical properties of resveratrol in peanut oil. Food Chem 368, 130687. 34416486 10.1016/j.foodchem.2021.130687

[129] Ahmadi R and Ebrahimzadeh MA (2020) Resveratrol – a comprehensive review of recent advances in anticancer drug design and development. Eur J Med Chem 200, 112356. 32485531 10.1016/j.ejmech.2020.112356

[130] Xu X , Liu A , Bai Y , Li Y , Zhang C , Cui S , Piao Y and Zhang S (2019) Co‐delivery of resveratrol and p 53 gene via peptide cationic liposomal nanocarrier for the synergistic treatment of cervical cancer and breast cancer cells. J Drug Deliv Sci Technol 51, 746–753.

[131] Ali MH , Moghaddam B , Kirby DJ , Mohammed AR and Perrie Y (2013) The role of lipid geometry in designing liposomes for the solubilisation of poorly water soluble drugs. Int J Pharm 453, 225–232. 22766442 10.1016/j.ijpharm.2012.06.056

[132] Szekeres T , Fritzer‐Szekeres M , Saiko P and Jäger W (2010) Resveratrol and resveratrol analogues—structure‐activity relationship. Pharm Res 27, 1042–1048. 20232118 10.1007/s11095-010-0090-1

[133] Queiroz AN , Gomes BAQ , Moraes WM Jr and Borges RS (2009) A theoretical antioxidant pharmacophore for resveratrol. Eur J Med Chem 44, 1644–1649. 18976835 10.1016/j.ejmech.2008.09.023

[134] Mikulski D , Górniak R and Molski M (2010) A theoretical study of the structure‐radical scavenging activity of trans‐resveratrol analogues and cis‐resveratrol in gas phase and water environment. Eur J Med Chem 45, 1015–1027. 20004046 10.1016/j.ejmech.2009.11.044

[135] Fang J‐G , Lu M , Chen Z‐H , Zhu H‐H , Li Y , Yang L , Wu L‐M and Liu Z‐L (2002) Antioxidant effects of resveratrol and its analogues against the free‐radical‐induced peroxidation of linoleic acid in micelles. Chemistry 8, 4191–4198. 12298009 10.1002/1521-3765(20020916)8:18<4191::AID-CHEM4191>3.0.CO;2-S

[136] Stivala LA , Savio M , Carafoli F , Perucca P , Bianchi L , Maga G , Forti L , Pagnoni UM , Albini A , Prosperi E *et al*. (2001) Specific structural determinants are responsible for the antioxidant activity and the cell cycle effects of resveratrol. J Biol Chem 276, 22586–22594. 11316812 10.1074/jbc.M101846200

[137] Shang Y‐J , Qian Y‐P , Liu X‐D , Dai F , Shang X‐L , Jia W‐Q , Liu Q , Fang J‐G and Zhou B (2009) Radical‐scavenging activity and mechanism of resveratrol‐oriented analogues: influence of the solvent, radical, and substitution. J Org Chem 74, 5025–5031. 19472994 10.1021/jo9007095

[138] Tang JJ , Fan G‐J , Dai F , Ding DJ , Wang Q , Lu DL , Li R‐R , Li X‐Z , Hu L‐M , Jin X‐L *et al*. (2011) Finding more active antioxidants and cancer chemoprevention agents by elongating the conjugated links of resveratrol. Free Radic Biol Med 50, 1447–1457. 21376113 10.1016/j.freeradbiomed.2011.02.028

[139] Ding D‐J , Cao X‐Y , Dai F , Li X‐Z , Liu G‐Y , Lin D , Fu X , Jin X‐L and Zhou B (2012) Synthesis and antioxidant activity of hydroxylated phenanthrenes as cis‐restricted resveratrol analogues. Food Chem 135, 1011–1019. 22953818 10.1016/j.foodchem.2012.05.074

[140] Yang J , Liu G‐Y , Lu D‐L , Dai F , Qian Y‐P , Jin X‐L and Zhou B (2010) Hybrid‐increased radical‐scavenging activity of resveratrol derivatives by incorporating a chroman moiety of vitamin e. Chemistry 16, 12808–12813. 20931567 10.1002/chem.201002020

[141] Szczepańska P , Rychlicka M , Groborz S , Kruszyńska A , Ledesma‐Amaro R , Rapak A , Gliszczyńska A and Lazar Z (2023) Studies on the anticancer and antioxidant activities of resveratrol and Long‐chain fatty acid esters. Int J Mol Sci 24, 7167. 37108328 10.3390/ijms24087167 PMC10139102

[142] Oh WY and Shahidi F (2017) Lipophilization of resveratrol and effects on antioxidant activities. J Agric Food Chem 65, 8617–8625. 28872859 10.1021/acs.jafc.7b03129

[143] Hu X‐P , Yin F‐W , Zhou D‐Y , Xie H‐K , Zhu B‐W , Ma X‐C , Tian X‐G , Wang C and Shahidi F (2019) Stability of resveratrol esters with caprylic acid during simulated in vitro gastrointestinal digestion. Food Chem 276, 675–679. 30409647 10.1016/j.foodchem.2018.10.062

[144] Neises B and Steglich W (1978) Simple method for the esterification of carboxylic acids. Angew Chem Int Ed Eng 17, 522–524.

[145] Tain Y‐L , Chang SKC , Liao J‐X , Chen Y‐W , Huang H‐T , Li Y‐L and Hou C‐Y (2021) Synthesis of short‐chain‐fatty‐acid resveratrol esters and their antioxidant properties. Antioxidants 10, 420. 33801821 10.3390/antiox10030420 PMC8001046

[146] Silva YP , Bernardi A and Frozza RL (2020) The role of short‐chain fatty acids from gut microbiota in gut‐brain communication. Front Endocrinol (Lausanne) 11, 25. 32082260 10.3389/fendo.2020.00025 PMC7005631

[147] Alrafas HR , Busbee PB , Nagarkatti M and Nagarkatti PS (2019) Resveratrol modulates the gut microbiota to prevent murine colitis development through induction of tregs and suppression of Th17 cells. J Leukoc Biol 106, 467–480. 30897248 10.1002/JLB.3A1218-476RR PMC6863607

[148] Liao J‐X , Chen Y‐W , Shih M‐K , Tain Y‐L , Yeh Y‐T , Chiu M‐H , Chang SKC and Hou C‐Y (2021) Resveratrol butyrate esters inhibit bpa‐induced liver damage in male offspring rats by modulating antioxidant capacity and gut microbiota. Int J Mol Sci 22, 5273. 34067838 10.3390/ijms22105273 PMC8156118

[149] Shih M‐K , Tain Y‐L , Cheng C‐M , Hsu C‐N , Chen Y‐W , Huang H‐T , Chang C‐I and Hou C‐Y (2021) Separation and identification of resveratrol butyrate Ester complexes and their bioactivity in HepG2 cell models. Int J Mol Sci 22, 13539. 34948341 10.3390/ijms222413539 PMC8703675

[150] Nègre‐Salvayre A , Augé N , Camaré C , Bacchetti T , Ferretti G and Salvayre R (2017) Dual signaling evoked by oxidized LDLs in vascular cells. Free Radic Biol Med 106, 118–133. 28189852 10.1016/j.freeradbiomed.2017.02.006

[151] ElAssar M , Angulo J and Rodríguez‐Mañas L (2013) Oxidative stress and vascular inflammation in aging. Free Radic Biol Med 65, 380–401. 23851032 10.1016/j.freeradbiomed.2013.07.003

[152] Imamura H , Nagayama D , Ishihara N , Tanaka S , Watanabe R , Watanabe Y , Sato Y , Yamaguchi T , Ban N , Kawana H *et al*. (2017) Resveratrol attenuates triglyceride accumulation associated with upregulation of Sirt 1 and lipoprotein lipase in 3T3‐L1 adipocytes. Mol Genet Metab Rep 12, 44–50. 28580300 10.1016/j.ymgmr.2017.05.003 PMC5448575

[153] Tang LY , Chen Y , Rui BB and Hu CM (2016) Resveratrol ameliorates lipid accumulation in Hep G2 cells, associated with down‐regulation of lipin 1 expression. Can J Physiol Pharmacol 94, 185–189. 26448098 10.1139/cjpp-2015-0125

[154] Shih M‐K , Hsieh S‐L , Huang Y‐W , Patel AK , Dong C and Hou C‐Y (2022) Resveratrol butyrate esters inhibit lipid biosynthesis in 3T3‐L1 cells by AMP‐activated protein kinase phosphorylation. J Food Sci Technol 60, 1015–1025. 36908355 10.1007/s13197-022-05436-x PMC9998790

[155] Traxinger BR , Richert‐Spuhler LE and Lund JM (2022) Mucosal tissue regulatory T cells are integral in balancing immunity and tolerance at portals of antigen entry. Mucosal Immunol 15, 398–407. 34845322 10.1038/s41385-021-00471-x PMC8628059

[156] Kim CH (2021) Control of lymphocyte functions by gut microbiota‐derived short‐chain fatty acids. Cell Mol Immunol 185, 1161–1171. 10.1038/s41423-020-00625-0 PMC8093302 33850311

[157] Liu P , Wang Y , Yang G , Zhang Q , Meng L , Xin Y and Jiang X (2021) The role of short‐chain fatty acids in intestinal barrier function, inflammation, oxidative stress, and colonic carcinogenesis. Pharmacol Res 165, 105420. 33434620 10.1016/j.phrs.2021.105420

[158] He J , Zhang P , Shen L , Niu L , Tan Y , Chen L , Zhao Y , Bai L , Hao X , Li X *et al*. (2020) Short‐chain fatty acids and their association with Signalling pathways in inflammation, glucose and lipid metabolism. Int J Mol Sci 21, 6356. 32887215 10.3390/ijms21176356 PMC7503625

[159] Krishnan S , Gertz ER , Adams SH , Newman JW , Pedersen TL , Keim NL and Bennett BJ (2022) Effects of a diet based on the dietary guidelines on vascular health and TMAO in women with cardiometabolic risk factors. Nutr Metab Cardiovasc Dis 32, 210–219. 34895998 10.1016/j.numecd.2021.09.013 PMC8798222

[160] Nguyen BO , Meems LMG , van Faassen M , Crijns HJGM , van Gelder IC , Kuipers F and Rienstra M (2021) Gut‐microbe derived TMAO and its association with more progressed forms of AF: results from the AF‐RISK study. Int J Cardiol Heart Vasc 34, 100798. 34095450 10.1016/j.ijcha.2021.100798 PMC8167185

[161] Winther SA , Øllgaard JC , Hansen TW , Von Scholten BJ , Reinhard H , Ahluwalia TS , Wang Z , Gæde P , Parving HH , Hazen S *et al*. (2021) Plasma trimethylamine N‐oxide and its metabolic precursors and risk of mortality, cardiovascular and renal disease in individuals with type 2‐diabetes and albuminuria. PLoS ONE 16, 1–14. 10.1371/journal.pone.0244402 PMC7928450 33657115

[162] Yao M‐E , Liao P‐D , Zhao X‐J and Wang L (2020) Trimethylamine‐N‐oxide has prognostic value in coronary heart disease: a meta‐analysis and dose‐response analysis. BMC Cardiovasc Disord 20, 7. 31918665 10.1186/s12872-019-01310-5 PMC6953212

[163] Marques BCAA , Trindade M , Aquino JCF , Cunha AR , Gismondi RO , Neves MF and Oigman W (2018) Beneficial effects of acute trans‐resveratrol supplementation in treated hypertensive patients with endothelial dysfunction. Clin Exp Hypertens 40, 218–223. 29431520 10.1080/10641963.2017.1288741

[164] Magyar K , Halmosi R , Palfi A , Feher G , Czopf L , Fulop A , Battyany I , Sumegi B , Toth K and Szabados E (2012) Cardioprotection by resveratrol: a human clinical trial in patients with stable coronary artery disease. Clin Hemorheol Microcirc 50, 179–187. 22240353 10.3233/CH-2011-1424

[165] Wong RHX , Berry NM , Coates AM , Buckley JD , Bryan J , Kunz I and Howe PRC (2013) Chronic resveratrol consumption improves brachial flow‐mediated dilatation in healthy obese adults. J Hypertens 31, 1819–1827. 23743811 10.1097/HJH.0b013e328362b9d6

